# Shared Genomic Regions Between Derivatives of a Large Segregating Population of Maize Identified Using Bulked Segregant Analysis Sequencing and Traditional Linkage Analysis

**DOI:** 10.1534/g3.115.017665

**Published:** 2015-06-01

**Authors:** Nicholas J. Haase, Timothy Beissinger, Candice N. Hirsch, Brieanne Vaillancourt, Shweta Deshpande, Kerrie Barry, C. Robin Buell, Shawn M. Kaeppler, Natalia de Leon

**Affiliations:** * Department of Agronomy, University of Wisconsin-Madison, Madison, Wisconsin 53706; ‡‡ Department of Energy Great Lakes Bioenergy Research Center, University of Wisconsin- Madison, Madison, Wisconsin 53706; † Department of Plant Sciences, University of California Davis, Davis, California 95616; ‡ Department of Agronomy and Plant Genetics, University of Minnesota, Saint Paul, Minnesota 55108; § Department of Plant Biology, Michigan State University, East Lansing, Michigan 48824; ** Department of Energy Great Lakes Bioenergy Research Center, Michigan State University, East Lansing, Michigan 48824; †† Department of Energy, Joint Genome Institute, Walnut Creek, California 94598

**Keywords:** quantitative trait analysis, maize, biomass, whole genome sequencing, genetic mapping

## Abstract

Delayed transition from the vegetative stage to the reproductive stage of development and increased plant height have been shown to increase biomass productivity in grasses. The goal of this project was to detect quantitative trait loci using extremes from a large synthetic population, as well as a related recombinant inbred line mapping population for these two traits. Ten thousand individuals from a B73 × Mo17 noninbred population intermated for 14 generations (IBM Syn14) were grown at a density of approximately 16,500 plants ha^−1^. Flowering time and plant height were measured within this population. DNA was pooled from the 46 most extreme individuals from each distributional tail for each of the traits measured and used in bulk segregant analysis (BSA) sequencing. Allelic divergence at each of the ∼1.1 million SNP loci was estimated as the difference in allele frequencies between the selected extremes. Additionally, 224 intermated B73 × Mo17 recombinant inbred lines were concomitantly grown at a similar density adjacent to the large synthetic population and were assessed for flowering time and plant height. Using the BSA sequencing method, 14 and 13 genomic regions were identified for flowering time and plant height, respectively. Linkage mapping with the RIL population identified eight and three regions for flowering time and plant height, respectively. Of the regions identified, three colocalized between the two populations for flowering time and two colocalized for plant height. This study demonstrates the utility of using BSA sequencing for the dissection of complex quantitative traits important for production of lignocellulosic ethanol.

Structural carbohydrates within maize stover have been proposed as an important biomass source for the fermentation process of sugars into lignocellulosic ethanol (Himmel *et al.* 2007; Lorenz and Coors 2008; Lorenz *et al.* 2009), an alternative to grain starch, which is currently and intensively used for ethanol production (Solomon *et al.* 2007; Yuan *et al.* 2008). Maize accounted for approximately 36.8 million of the hectares planted in the United States in 2013, with 33.6 million hectares being harvested for grain production alone (USDA 2014). In a recent report from the United States Department of Energy (DOE), maize crop residues accounted for an estimated 70% of the annual grain crop residues from 1998 to 2007 (Perlack and Stokes 2011). Therefore, increasing the amount of corn stover biomass yield would have value in supporting the emerging lignocellulosic biofuel industry.

Plant height (PH) is positively correlated with biomass yield in maize and sorghum (Lubberstedt *et al.* 1997; Murray *et al.* 2008; Ritter *et al.* 2008). The correlation between flowering time (FT) and PH, as well the correlation of FT with other morphological traits related to above-ground biomass production such as total leaf number through the timing of vegetative to reproductive transition in maize, suggests that FT has the potential to impact biomass yield (Irish and Nelson 1991; Yuan *et al.* 2008). Due to this relationship, both PH and FT were chosen as model traits for this study. Furthermore, understanding independent genetic regions that are responsible for these two traits could ultimately help develop higher biomass yielding maize varieties while maintaining appropriate ranges of maturity by ensuring that changes in plant height would not greatly affect the flowering time of an individual.

PH and FT are extensively studied phenotypic traits in maize. Although these traits are relatively highly heritable, it is likely that only a fraction of the genomic regions contributing to their variation are currently known. Traditional linkage mapping studies have identified 5–12 (6.7 average) and 1–12 (4.6 average) quantitative trait loci (QTL) associated with PH and FT (silking date and days to anthesis), respectively (Austin and Lee 1996; Beavis *et al.* 1991; Bohn *et al.* 2000; Cardinal *et al.* 2001; Chardon *et al.* 2004; Tang *et al.* 2007; Zhang *et al.* 2011). Larger mapping populations, such as the United States Nested Association Mapping (US-NAM) population, have uncovered numerous small to moderate effect QTL and provide a more detailed dissection of the genetic architecture of these complex traits compared to the small number QTL commonly observed in traditional linkage populations (Buckler *et al.* 2009; Peiffer *et al.* 2014). However, phenotyping and genotyping such large collections can be both labor-intensive and expensive to conduct. Bulk segregant analysis (BSA) using whole genome sequencing data has been proposed as a method that can be used to identify QTL for genetically complex traits (Ehrenreich *et al.* 2010).

BSA was originally proposed by Michelmore *et al.* (1991) to rapidly identify markers linked to particular traits of interest. Their approach involves a segregating F_2_ population generated from an initial cross between two phenotypically diverse parents, which is then scored for a phenotype of interest. Bulked DNA or RNA samples are constructed from individuals that show contrasting phenotypes. Genetic markers are then used to screen for differences between the two DNA or RNA pools that associate with the trait of interest. BSA has been mainly used in crop species either for the identification of large effect QTL, such as disease resistance genes, or for mapping qualitative mutations (Quarrie *et al.* 1999; Hyten *et al.* 2009; Venuprasad *et al.* 2009; Liu *et al.* 2012).

The availability of high-density genotyping technologies (Metzker 2010) have allowed for the rapid identification of single nucleotide polymorphisms (SNPs) that may be associated with phenotypes of interest, thereby increasing the ability to identify causative regions controlling important traits. However, the cost of sequencing entire populations can still be relatively high, and therefore is still not economically feasible for the assessment of large numbers of recombinant progenies. Genome reduction methods such as genotype-by-sequencing (GBS) can help reduce the cost of genotyping the large numbers of required individuals (Elshire *et al.* 2011). However, these technologies also result in a high proportion of missing information (Beissinger *et al.* 2013).

Approaches that use whole genome sequencing of bulked pools of DNA have been used to identify QTL or selected regions in model organisms, such as yeast (*Saccharomyces cerevisiae*) and *Drosophila* (*Drosophila melanogaster*) (Ehrenreich *et al.* 2010; Magwene *et al.* 2011; Turner *et al.* 2011). These model organisms allow for the generation of very large populations of segregating individuals. This approach has similarly been used for the identification of QTL in rice (Takagi *et al.* 2013). When applied to a rice RIL population, colocalization of the most significant QTL for resistance to rice blast was observed between linkage mapping and whole genome sequencing on bulked samples of extremes (Takagi *et al.* 2013). Additionally, this study demonstrated that this method has the ability to detect QTL for important agronomic traits, such as seedling vigor, using an F_2_-derived rice population, of which some regions identified colocalized with previously reported QTL from other mapping studies (Miura *et al.* 2001; Fujino *et al.* 2008; Takagi *et al.* 2013).

The primary aim of this study was to use BSA sequencing in conjunction with linkage mapping information to identify QTL for two quantitative traits, PH and FT, important for producing lignocellulosic ethanol. This was accomplished using phenotypic extremes from a large segregating synthetic maize population grown concomitantly with a related RIL population.

## Materials and Methods

### Plant materials

The intermated B73 × Mo17 (IBM) Syn14 population was used for the BSA sequencing analysis. This population was derived from intermating the progenitor population, the IBM Syn10, for four additional generations. The IBM Syn10 was derived by intermating the F_2_ generation from the initial cross of maize inbred lines B73 and Mo17 for 10 generations (Hussain *et al.* 2007). For the QTL analysis, 224 lines from the IBM RIL population were used (see Supporting Information, Table S1 and Table S2; Lee *et al.* 2002). These RILs were derived by intermating the F_2_ generation from the initial cross of B73 × Mo17 for four generations before starting the process of selfing.

### Phenotypic collection and analysis

Ten thousand segregating variants from the IBM Syn14 population were planted in 2011 at the West Madison Agricultural Research station in Madison, Wisconsin. To minimize plant-to-plant competition, a planting density of approximately 16,500 plants ha^−1^ was used. Plants were distributed in the field at a distance of 0.76 meters on all sides of each plant. The IBM RILs were planted at the same density as the IBM Syn14 population in a randomized complete block design (RCBD) using two replications at that location. Additionally, the IBM RILs were also planted at a density of approximately 49,000 plants ha^−1^ in an RCBD at the same location with two replications. The IBM RILs grown at the two different densities (16,500 and 49,000 plants ha^−1^) are referred to herein as the IBM density trial.

For individual plants, FT was determined as the first day in which 50% of the tassel spike was exerting anthers. Using information available on cumulative growing degree days (GDD) for Madison, provided by the State of Wisconsin Department of Administration, this measurement was then converted to GDD to pollen shed, and was only recorded for the 226 earliest and 112 latest flowering individuals in the population during the summer of 2011. PH, measured as the distance (cm) from the soil surface to the flag leaf ligule, was also collected. Only the 154 shortest and 158 tallest plants were recorded. The 46 most extreme individuals from each tail of the distribution were then selected for each trait measured (Table S3). PH and FT were also determined on a plot basis for all IBM RILs grown using two different planting densities. FT was recorded when half of the plot flowered, according to the method outlined above. Additionally, PH was measured (as described above) on five healthy plants from each plot, and plot means were calculated as the experimental unit for analysis.

Phenotypic data from the IBM density trial were analyzed using SAS PROC MIXED version 9.2 (SAS Institute) with the following mixed linear model:

$$Y_{ijk}=\mu +d_{i}+R{\left(D\right)}_{j}+G_{k}+GD_{ik}+\varepsilon_{ijk}$$ [1]

where Y_ijk_ is the response variable of the k^th^ genotype (*G*) in the j^th^ replicate (*R*) nested in the i^th^ density (*D*). The residual error ε_ijk_ was assumed to be independent and following a normal distribution (∼iidN(0,$\sigma_{\varepsilon }^{2}$)). Genotype, replicate, and error were considered random effects, whereas density was considered to be a fixed effect. Additionally, best linear unbiased predictions (BLUPs), to be used for QTL mapping, were calculated for each genotype in both densities separately using equation [1], removing density and genotype-by-density from the model.

### DNA extraction and sequencing

In the IBM Syn14 population, leaf tissue was collected from 92 random immature plants to be used as a control group. Inner husk tissue was also collected from the 46 most extreme plants from each distributional tail for both traits measured. Genomic DNA was extracted for individual samples using a modified CTAB method (Sanghai-Maroof *et al.* 1984). Equimolar DNA pools were then constructed from 46 extreme individuals for each distributional tail (*i.e.*, early flowering, late flowering, tall PH, and short PH) and the 92 random control plants.

Libraries with a target insert size of 500 bp were prepared according to the Illumina protocol (Illumina, Inc., San Diego, CA). Libraries were sequenced using the Illumina HiSequation 2000 (San Diego, CA) at the Joint Genome Institute (Walnut Creek, CA) to generate 100 nt paired-end sequence reads for the early flowering pool and 150 nt paired-end sequence reads for all other pools. Sequence read quality was evaluated using the FastQC program (http://www.bioinformatics.babraham.ac.uk/projects/fastqc/).

### Generation of a Mo17 reference sequence

To generate a reference genome for Mo17 and thereby reduce bias in read mapping, genomic reads were cleaned using the FASTX toolkit (http://hannonlab.cshl.edu/fastx_toolkit/index.html) prior to mapping. The fastx_clipper program was used to remove the Illumina paired-end adapter sequences requiring a minimum sequence length of 15 nt after clipping. Sequence reads were quality trimmed using the fastq_quality_trimmer requiring a minimum quality score of 20 and a minimum read length of 15 nt. All reads that passed through the cleaning step above were mapped as single-end reads using Bowtie version 0.12.7 (Langmead *et al.* 2009) to the B73 v2 reference sequence (Schnable *et al.* 2009). An alignment was considered valid if there were two or fewer mismatches relative to the reference sequence (-v 2) and a read was required to have only one valid alignment (-m 1). All other parameters were set to the default values.

Alignment files from all five pools were processed together using the sort, merge, index, and pileup programs within SAMtools version 0.1.12a (Li *et al.* 2009) to generate a single unfiltered pileup file. For the pileup program, the -B option was used to disable BAQ computation. Single nucleotide polymorphisms (SNPs) relative to the B73 reference assembly were identified for positions with at least 10× coverage using only bases from reads with a quality score of 20 or more and requiring a minimum allele frequency of 0.25. In total, 3,301,371 SNPs were called relative to the B73 reference sequence. The corrected Mo17 reference sequence was generated by substituting the alternative allele at all polymorphic positions.

### Estimating B73 and Mo17 allele frequencies

To reduce bias between the pools due to sequence length, reads from the five pools were further cleaned using the fastx_trimmer program within the FASTX toolkit (http://hannonlab.cshl.edu/fastx_toolkit/index.html), allowing a maximum sequence length of 100 nt. Reads were simultaneously mapped as single-end reads to both the B73 v2 reference sequence as well as our Mo17 reference sequence using Bowtie version 0.12.7 (Langmead *et al.* 2009) requiring a perfect match (-v 0) and a unique alignment (-m 1). Only sequence reads that mapped to either the B73 or the Mo17 reference sequence, but not both, were retained. Alignments from the retained reads were processed using the sort, merge, index, and pileup programs within SAMtools version 0.1.12a (Li *et al.* 2009) to generate an unfiltered pileup file for each of the five pools. Allele frequency estimates within each pool were determined for the B73 and Mo17 alleles at the 3,301,371 previously identified SNP loci. Only reads with a quality score of 20 or more were used to estimate allele frequencies. To obtain accurate allele frequency estimates, if the coverage within a sequenced pool was less than 20 or greater than 60.8 (mean across the pools plus 1 SD) the position was considered missing data within that pool. Finally, positions that had a B73 allele frequency of less than 0.25, greater than 0.75, or missing data in the control population were discarded. After filtering, 1,096,729 and 1,149,984 polymorphic SNPs were retained for further analysis for FT and PH, respectively. The observed genome-wide mean for the estimated B73 allele frequency was 0.48±0.18 for early, 0.52±0.15 for late, 0.53±0.18 for short, 0.52±0.19 for tall, and 0.53±0.13 for control pools.

### BSA sequencing and QTL mapping

B73 allele frequencies for each locus were estimated using the read counts in each of the five pools as described above. Using a custom script written in R version 3.1.1 (File S1; R Core Team 2014) using the zoo software package (Zeileis and Grothendieck 2005), a standard two-sided Z-test was then performed to determine the significance of each difference in terms of allele frequency between the pools of extreme individuals for each loci tested for both FT and PH and was allowed to slide over windows such that

$$Z^{\prime }=\;\frac{1}{d}\overset{d}{\underset{j=1}{\sum }}\frac{p_{j(top)}-p_{j(bottom)}}{\sqrt{{\hat{p}}_{j}\left(1-{\hat{p}}_{j}\right)*(\frac{1}{n_{j\left(top\right)}}\;+\;\frac{1}{n_{j(bottom)}})}}$$ ; [2]

$${\hat{p}}_{j}=\;\frac{x_{j\left(top\right)}+x_{j\left(bottom\right)}}{n_{j(top)}+n_{j(bottom)}}\;$$ [3]

where p_j(top)_ and p_j(bottom)_ were the estimated allele frequencies and n_j(top)_ and n_j(bottom)_ were the observed number of reads for the j^th^ SNP between the two pools for the two traits measured (*i.e.*, early minus late FT, or tall minus short PH plants). The expected allele frequency, ${\hat{p}}_{j}$, was calculated using the number of reads for the B73 allele (x_j(top)_ and x_j(bottom)_) and the total number of observed reads (n_j(top)_ and n_j(bottom)_) for the j^th^ SNP. This statistic was then averaged across a window of size d, which was equal to 15 SNPs, and then compared back to a standardized normal distribution to obtain p-values for each SNP. The negative log_10_ of the p-values were then used for identifying significant SNPs. Because statistics based on windows of several markers were applied across regions of unknown linkage disequilibrium between groups of markers, creating permutation thresholds was not feasible for this study. Likewise, a Bonferroni correction was too conservative and FDR thresholds (Benjamini and Hochberg 1995) were generally too liberal. Therefore, to correct for multiple testing, an outlier threshold of 0.5% used for the calling of significant QTL was considered the most reasonable approach. This approach was similar to those used for detecting selective sweeps using pooled DNA samples for unidirectional and divergently selected populations of maize (Beissinger *et al.* 2014; Hirsch *et al.* 2014). The boundary of a significant region included all SNPs with a –log_10_(p-value) over this threshold and the adjacent seven SNPs upstream and downstream of the significant region, as these markers were used to estimate the last significant SNPs within a region. To resolve peaks, a nonsignificant region of 5 Mb was required between the left-most SNP of a significant region and the right-most SNP of the prior significant region. This approach was also similar to that used by Beissinger *et al.* (2014) while using whole genome sequencing on bulked samples for scanning selection sites in a population of maize undergoing recurrent selection for prolificacy.

QTL mapping was performed separately on the IBM data for both densities (16,500 plants ha^−1^ and 49,000 plants ha^−1^) from the summer of 2011 for both PH and FT. BLUPs for each genotype, used for mapping, were calculated from a linear model for each density separately. Mapping was performed on the marker set outlined and provided in the work by Burton *et al.* (2015). Using the software SEG-Map (Zhao *et al.* 2010), the authors imputed a parental phase for markers generated from GBS to generate a total of 8224 recombination bin breakpoints that were used as markers (Burton *et al.* 2015). Additionally outputted from SEG-Map was a conversion file for genetic to physical positions on all estimated breakpoints. QTL mapping was conducted using the stepwise multiple QTL mapping function in the software program R/qtl (Broman *et al.* 2003). A permuted LOD threshold was determined for each trait mapped using 1000 permutations of a single QTL model with no covariates. LOD intervals of 1.5 were then used to define the boundaries of significant regions. Mapping was conducted using genetic positions, which were converted to physical positions using the IBM map conversion file for comparison to the BSA sequencing method.

## Results

### Phenotypic evaluation

**Figure 1:**
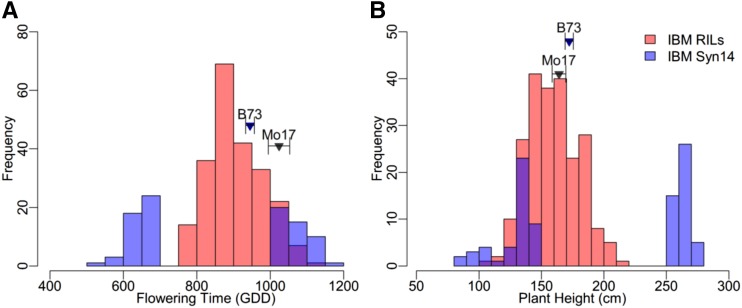
Phenotypic distributions for both measured traits. Distributions are shown for both the intermated B73 × Mo17 (IBM) recombinant inbred line (RIL) and Syn14 populations. Distributions for the IBM RILs are for one trial grown in the summer of 2011 at approximately 16,500 plants ha^−1^ and are averaged across two replicates. Distributions for the IBM Syn14 population include the 92 selected extreme individuals flowering time in (A) growing degree days (GDD) and (B) plant height. Purple indicates areas where the distributions overlap. Parental values for B73 and Mo17 are indicated by blue and black arrows, respectively.

Phenotypic distributions for the IBM Syn14 population and IBM RILs, both grown at low density, were compared for FT and PH (Figure 1). Both traits showed larger amounts of transgressive segregation of phenotypes within the IBM Syn14 population relative to the IBM RILs, which is expected given the much greater number of individuals in the Syn14 population. PH varied from 109 to 211 cm in the IBM RILs and from 85 to 280 cm in the IBM Syn14 population. Additionally, the mean of the 92 (46 from each phenotypic tail) selected individuals in the IBM Syn14 population (198 cm), was shifted toward taller individuals relative to the IBM RILs (159 cm). This is consistent with dominance and heterosis for PH given that, on average, ∼50% of the loci will be heterozygous in the individual Syn14 plants, whereas the IBM RILs are highly inbred. FT varied from 751 to 1103 GDD in the IBM RILs and from 528 to 1158 GDD in the IBM Syn14 population. Similarly, the 92 selected individuals from the IBM Syn14 population showed a shift in the mean toward earliness (858 GDD) relative to the IBM RIL (906 GDD). This observation is also consistent with dominance and heterosis for FT.

Significant genetic variation was observed for the IBM lines grown in the IBM density trial for both traits measured (ANOVA, *P* < 2×10^−16^). However, a significant genotype × density interaction was also observed for FT (ANOVA, *P* = 0.03).

### QTL detection in extreme individuals from the synthetic heterogeneous population

**Figure 2:**
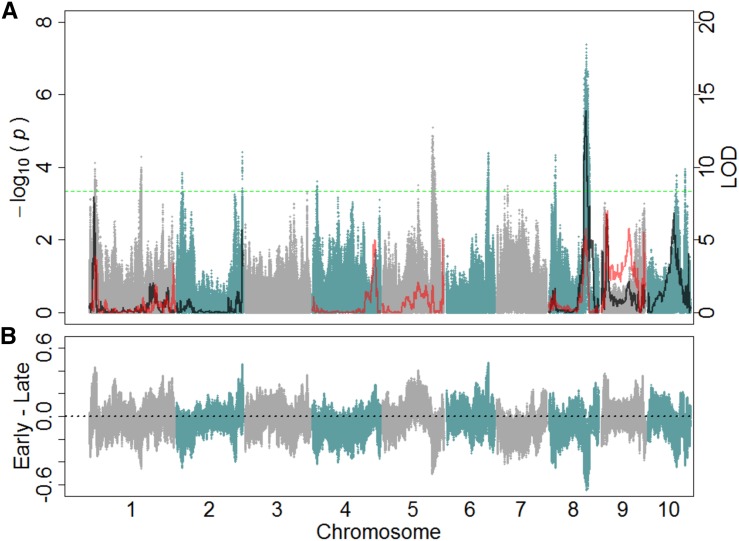
Genetic mapping of flowering time. (A) Shown are both bulk segregant analysis (BSA) sequencing and traditional linkage quantitative trait loci mapping methods for flowering time. Blue and gray profile, corresponding to the left y-axis, was from the analysis of frequency differences between extreme pools (BSA sequencing). Each single nucleotide polymorphism (SNP) position is estimated using supporting information from the 14 neighboring SNPs. The green dotted line indicates a 0.5% empirical outlier threshold for the BSA sequencing. Black (16,500 plants ha^−1^) and red (49,000 plants ha^−1^) LOD profiles, corresponding to the right y-axis, show traditional linkage mapping results in the intermated B73 × Mo17 (IBM) recombinant inbred lines (RILs) determined using R/qtl. Only chromosomes containing significant associations are displayed (LOD >3.61). (B) Differences in the B73 allele frequency between the early and late pools are shown. Each SNP position is estimated using supporting information from the 14 neighboring SNPs.

Using the BSA sequencing method in the IBM Syn14 population with a genome-wide significance threshold of 3.35, a total of 14 regions were identified to be significantly associated with FT (Figure 2A; Table 1). The two most significant regions found for FT were 10.8 Mb and 18.9 Mb in size and located on chromosomes 5 and 8, respectively. These regions were also the largest genomic regions identified. The Mo17 allele conferred earliness for 10 out of the 14 regions, one of which was the most significant region (second region on chromosome 8; Figure 2B).

**Table 1:** Significant flowering time regions in intermated B73 × Mo17 (IBM) Syn14 and IBM recombinant inbred lines

Chromosome	Left Position	Right Position	Most Significant Position	Length (kb)	Density (Plants ha^−1^)	p-value	Average Allelic Effect ([Mo17-B73]/2)*c*	% Variation Explained	Method
1*^a^*,*^b^*	11,100,000	21,850,000	13,450,000	10,750	49,000	1.18e-5	13.7	4.58	LM
1*^a^*,*^b^*	15,150,000	19,050,000	15,950,000	3900	16,500	6.71e-10	18.4	8.27	LM
1*^a^*,*^b^*	17,714,079	22,596,124	18,463,651	4882	16,500	7.65e-5	(+)	—	BSAS
1	180,711,478	183,050,137	181,687,511	2339	16,500	5.06e-5	(−)	—	BSAS
2	19,435,989	19,451,856	19,443,208	16	16,500	1.44e-4	(−)	—	BSAS
2*^a^*	233,150,000	234,950,000	234,650,000	1800	16,500	1.42e-7	15.5	5.79	LM
2*^a^*	233,684,368	234,219,869	234,209,964	536	16,500	3.85e-5	(+)	—	BSAS
4	14,822,931	17,108,700	16,180,806	2286	16,500	2.39e-4	(−)	—	BSAS
4	216,050,000	225,100,000	223,100,000	9050	49,000	7.95e-7	16.5	5.98	LM
5	127,205,233	127,239,082	127,215,227	34	16,500	3.09e-4	(+)	—	BSAS
5	175,599,023	186,393,293	178,591,431	10,794	16,500	7.97e-6	(−)	—	BSAS
5	212,950,000	214,450,000	214,150,000	1500	49,000	6.37e-7	17.1	6.09	LM
6	146,682,333	147,926,947	146,815,395	1245	16,500	3.92e-5	(+)	—	BSAS
7	27,603,027	27,613,362	27,607,683	10	16,500	4.22e-4	(−)	—	BSAS
7	39,302,108	39,309,960	39,306,816	8	16,500	3.20e-4	(−)	—	BSAS
8	18,487,954	21,420,111	21,411,057	2932	16,500	4.69e-5	(−)	—	BSAS
8*^a^*,*^b^*	123,504,621	142,361,278	131,086,800	18,857	16,500	4.13e-8	(−)	—	BSAS
8*^a^*,*^b^*	124,350,000	134,700,000	131,250,000	10,350	49,000	1.11e-7	−18.3	7.02	LM
8*^a^*,*^b^*	127,700,000	133,050,000	131,250,000	5350	16,500	6.94e-16	−30.7	15.29	LM
8	143,700,000	144,300,000	144,050,000	600	16,500	3.07e-9	20.8	7.56	LM
9	3,750,000	6,400,000	5,250,000	2650	16,500	4.92e-6	13.7	4.23	LM
9*b*	17,650,000	24,350,000	22,600,000	6700	49,000	6.02e-9	19.2	8.61	LM
9*b*	18,650,000	24,250,000	20,550,000	5600	16,500	1.15e-8	17.2	6.94	LM
9	152,250,000	153,550,000	152,750,000	1300	49,000	2.23e-7	−16.8	6.65	LM
10	87,300,000	94,200,000	92,600,000	6900	16,500	9.07e-9	−17.1	7.05	LM
10	99,225,452	102,316,547	102,314,009	3091	16,500	1.61e-4	(−)	—	BSAS
10	132,510,585	132,547,745	132,528,197	37	16,500	1.12e-4	(−)	—	BSAS
10	144,950,000	146,250,000	146,050,000	1300	16,500	7.17e-6	−12.8	4.07	LM

Regions were identified using the bulk segregant analysis sequencing (BSAS) method using 92 phenotypically extreme individuals from the intermated B73 × Mo17 (IBM) Syn14 population and linkage mapping (LM) with 8224 bin markers for 224 IBM recombinant inbred lines grown in two replications at densities of approximately 16,500 and 49,000 plants ha^−1^ in one environment. Also included are the left and right boundary positions, the most significant marker position, size of the interval, p-value of the most significant position, estimated QTL effect, and percent variation explained by each RIL QTL.

a Shared regions between the two populations.

b Shared regions between densities.

c Only directionality of allele frequency shift is reported for regions identified by BSAS in the IBM Syn14.

**Figure 3:**
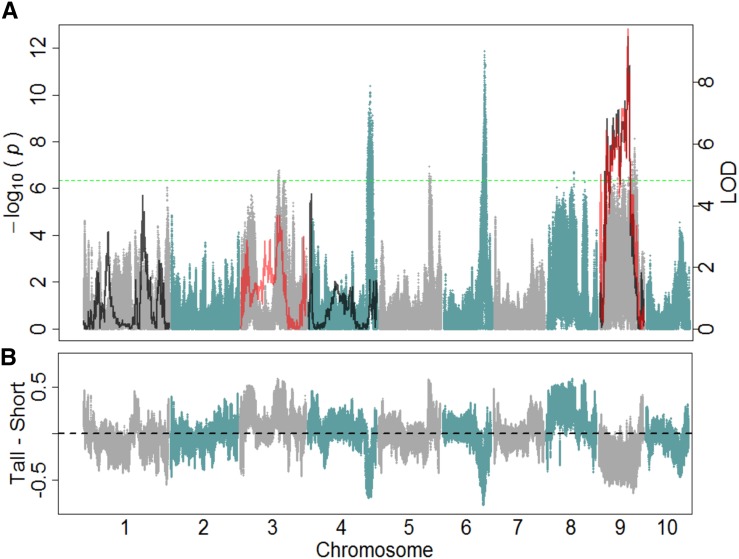
Genetic mapping of plant height. (A) Shown are both bulk segregant analysis (BSA) sequencing and traditional linkage quantitative trait loci mapping methods for plant height. Blue and gray profile, corresponding to the left y-axis, was from analysis of frequency differences between extreme pools (BSA sequencing). Each single nucleotide polymorphism (SNP) position is estimated using supporting information from the 14 neighboring SNPs. The green dotted line indicates a 0.5% empirical outlier threshold for the BSA sequencing. Black (16,500 plants ha^−1^) and red (49,000 plants ha^−1^) LOD profiles, corresponding to right y-axis, show linkage mapping results in the intermated B73 × Mo17 (IBM) recombinant inbred lines (RILs) determined using R/qtl. Only chromosomes containing significant associations are displayed (LOD >3.68). (B) Differences in the B73 allele frequency between the tall and short pools are shown. Each SNP position is estimated using supporting information from the 14 neighboring SNPs.

The BSA sequencing method was also applied to the IBM Syn14 population for PH. A total of 13 regions were identified to be associated with PH at a significance threshold of 6.34 (Figure 3A; Table 2). The two most significant regions found for PH were 21.2 Mb and 9.7 Mb in size and located on chromosomes 4 and 6, respectively. Seven of the 13 regions were located on chromosome 9 alone. Of the regions identified, B73 conferred the tall allele for 4 out of the 13 regions (Figure 3B). However, for the three most significant regions located on chromosomes 4, 6, and 9, Mo17 contributed the tall allele.

**Table 2:** Significant plant height regions in intermated B73 × Mo17 (IBM) Syn14 and IBM recombinant inbred lines

Chromosome	Left Position	Right Position	Most Significant Position	Length (kb)	Density (Plants ha^−1^)	p-value	Average Allelic Effect ([Mo17-B73]/2)*c*	% Variation Explained	Method
1	202,350,000	207,100,000	206,250,000	4750	16,500	3.93e-6	4.8	6.57	LM
3*^a^*,*^b^*	124,500,000	149,550,000	129,050,000	25,050	49,000	1.88e-5	−4.8	5.69	LM
3*^a^,^b^*	131,761,170	133,787,782	133,783,580	2027	16,500	1.62e-7	(−)	—	BSAS
4	5,650,000	10,550,000	9,850,000	4900	16,500	3.49e-6	4.9	6.65	LM
4	205,171,472	226,328,731	215,273,358	21,157	16,500	4.24e-11	(+)	—	BSAS
5	175,750,134	175,756,910	175,754,067	7	16,500	1.18e-7	(−)	—	BSAS
5	180,878,329	180,885,452	180,882,909	7	16,500	3.11e-7	(−)	—	BSAS
6	138,185,595	147,926,947	143,999,660	9741	16,500	1.39e-12	(+)	—	BSAS
8	92,560,823	92,576,189	92,568,159	15	16,500	1.92e-7	(−)	—	BSAS
9	4,950,000	5,650,000	5,250,000	700	49,000	7.36e-7	5.7	7.87	LM
9	38,353,868	38,359,043	38,357,148	5	16,500	3.18e-7	(+)	—	BSAS
9	61,337,181	61,343,573	61,340,803	6	16,500	3.54e-7	(+)	—	BSAS
9	81,804,594	82,892,514	81,814,361	1088	16,500	4.25e-7	(+)	—	BSAS
9*^a^*,*^b^*	96,000,000	105,050,000	99,050,000	9050	16,500	1.89e-11	7.2	15.19	LM
9*^a^*,*^b^*	96,450,000	105,050,000	99,050,000	8600	49,000	1.08e-11	7.7	16	LM
9*^a^*,*^b^*	100,914,404	103,222,106	100,917,919	2308	16,500	3.36e-7	(+)	—	BSAS
9	113,791,080	114,932,420	114,276,702	1141	16,500	7.11e-8	(+)	—	BSAS
9	121,580,239	125,181,457	122,087,889	3601	16,500	7.3e-9	(+)	—	BSAS
9	130,719,322	130,920,301	130,885,891	201	16,500	1.67e-7	(+)	—	BSAS

Regions were identified using the bulk segregant analysis sequencing (BSAS) method using 92 phenotypically extreme individuals from the intermated B73 × Mo17 (IBM) Syn14 population and linkage mapping (LM) with 8224 bin markers for 224 IBM recombinant inbred lines grown in two replications at densities of approximately 16,500 and 49,000 plants ha−1 in one environment. Also included are the left and right boundary positions, the most significant marker position, size of the region, p-value of the most significant position, estimated QTL effect, and percent variation explained by each RIL QTL.

a Shared regions between the two populations.

b Shared regions between densities.

c Only directionality of allele frequency shift is reported for regions identified by BSAS in the IBM Syn14.

### QTL detection using linkage mapping with RILs

Data from the low density (approximately 16,500 plants ha^−1^) planting of the IBM RILs in 2011 were used for the detection of QTL for both FT and PH. Eight regions were identified for FT (LOD > 3.67; Figure 2A) on chromosomes 1, 2, 8, 9, and 10, with the most significant region being located on chromosome 8. Genetic positions were converted to physical positions to determine the relative size for each of the regions identified (Table 1). The size of the 1.5 LOD intervals for these regions varied in size from 600 kb to 6.9 Mb in length. The estimated parental effects of B73 for five of the eight regions identified were toward earliness. Additionally, three QTL were identified for PH (LOD > 3.7; Figure 3A) on chromosomes 1, 4, and 9, with the most significant region located on chromosome 9. The 1.5 LOD intervals for these identified regions varied from 4.9 Mb to 9.1 Mb (Table 2). The estimated parental effects of B73 for the three regions identified were toward shorter plants. Despite the relatively high heritability of the two traits measured, the QTL model only explained 54% and 30% of the variation for FT and PH, respectively.

Data from the high density (approximately 49,000 plants ha^−1^) planting of the IBM RILs in 2011 were also used for the detection of QTL for both FT and PH. A total of six regions were identified for FT (LOD > 3.66) located on chromosomes 1, 4, 5, 8, and 9. The 1.5 LOD intervals for these regions varied from 1.3 Mb to 10.8 Mb in length (Table 1; Figure 2A). The estimated parental effects of B73 for four of the six regions identified were toward earliness. Additionally, of the six regions identified, QTL located on chromosomes 1 (11.1 Mb–21.85 Mb), 8 (124.35 Mb–131.25 Mb), and 9 (17.65 Mb–24.35 Mb) coincided with QTL identified in the low-density treatment (Table 1) and shared the same directionality of estimated effects. A total of three regions were identified for PH (LOD > 3.62) located on chromosomes 3 and 9, with 1.5 LOD intervals ranging from 700 kb to 25.1 Mb in length (Table 2; Figure 3A). The estimated parental effects of B73 for one of the three regions identified were toward taller plants. Additionally, one QTL located on chromosome 9 (96.45 Mb–105.05 Mb) coincided with QTL identified in the low-density treatment (Table 2) and shared the same directionality of estimated effects.

### Overlapping QTL region analysis

Overlapping regions between QTL detected in the Syn14 and low-density IBM set were declared if the physical positions of a 1.5 LOD interval from the traditional linkage mapping in the IBM RIL population fell within the boundaries of a region identified using the BSA sequencing method. Based on the physical position of the 1.5 LOD interval and the boundaries of the BSA sequencing method overlap, three regions located on chromosomes 1, 2, and 8 were identified for FT. The 1.5 LOD interval for linkage mapping extended into the significant region identified using BSA sequencing on chromosome 1. The 1.5 LOD interval for linkage mapping was contained within the significant region identified using BSA sequencing for chromosome 8, whereas the 1.5 LOD interval encompassed the BSA sequencing region on chromosome 2. The estimated effects for the IBM RIL QTL regions were consistent with the individual conferring the early or late allele in the IBM Syn14.

Two regions were identified as an overlapping region for PH. The QTL identified on chromosome 9 using linkage mapping in the 16,500 plants ha^−1^ treatment fell within the fourth region identified on chromosome 9 (100.9–103.22 Mb) using the BSA sequencing method. None of the other 12 regions identified using the Syn14 coincided with QTL found using traditional linkage mapping in the IBM RILs at this density. There was an additional overlap found between the 49,000 plant ha^−1^ IBM treatment and the IBM Syn14 on chromosome 3 (125–150 Mb). As with flowering time, the directionality of the estimated effects for the IBM QTL was consistent with the individual conferring the short or tall parental allele in the IBM Syn14.

## Discussion

This study used a BSA sequencing approach to identify QTL for FT and PH in a large synthetic population. When compared to QTL from the IBM RIL population derived from the same parents, three regions of concordance were observed between the two populations for FT and two for PH. The BSA sequencing on the Syn14 population identified a larger number of QTL for FT and PH relative to QTL identified in the RIL population. It has been shown that in instances where a trait is highly polygenic with moderate effects, larger mapping populations are beneficial to increase statistical power and prevent overestimation of QTL effects (Beavis 1998; Xu 2003; Broman 2001). The population size of the IBM Syn14 relative to IBM RILs could have potentially increased the power to detect additional moderate effect QTL. This has also been shown through power analysis simulations examining the relative effect of population size on QTL detection using BSA (Magwene *et al.* 2011). Additionally, in populations that have higher numbers of recombination between genotypes, it is expected that repulsion and coupling phase linkages will be broken, allowing for greater power to detect QTL. In the IBM RIL population there are, on average, 57 effective recombination events per individual (Fu *et al.* 2006). Using the known average effective recombination events in the IBM RIL population and the expansion equations *x[j/2 + (2^i^−1)/2^i^]* and *x[(2^i+1^ −1)/2^i^]* outlined by Beissinger *et al.* (2013), where *i* is the number of generations a line has been inbred and *j* is the number of generations of intermating, we can estimate the average effective recombination events to be approximately 152 per individual in the IBM Syn14 population (Beissinger *et al.* 2013; Teuscher *et al.* 2005). Thus, the effective recombination in the IBM Syn14 population is more than twice the amount observed in the RILs. These considerations are consistent with recent studies using the maize US-NAM population and a large association panel. Coupling the use of large populations with the ability to utilize historical recombination, these studies elucidated the highly polygenic nature of PH and FT (Buckler *et al.* 2009; Peiffer *et al.* 2014).

In addition to QTL regions identified in the IBM Syn14 population not being identified in the IBM RIL population, the inverse was also observed. This was contrary to the expected result that all regions identified in the IBM RILs would have been detected using the larger IBM Syn14 population due to the prior considerations discussed. Although a larger population was grown, a relatively small portion of the allelic variation was sampled and used for conducting statistical tests. This is consistent with power analysis simulations conducted by Magwene and Colleagues (2011) examining at the effects of sample size and sequencing coverage on the detection of expected QTL. In these simulations, population and sample sizes and sequencing coverage of magnitude similar to the parameters used in this study resulted in among the lowest power to detect QTL. It is possible that the observed result was caused by a sampling effect generated by using the higher selection intensity chosen (∼0.5% selected in each tail out of 10,000 plants). This observation is primarily a sampling issue in that the individuals being used for the statistical test is a small sample of the substantially larger population one is trying to describe (Beavis 1998; Xu 2003). Using sample sizes of one order of magnitude larger is expected to have yielded the detection of more QTL within the IBM Syn14 population.

Although the lower planting density used for the IBM Syn14 was originally chosen to mitigate interplant competition between individuals measured, it was observed that genotype × density interactions existed for FT in the IBM RIL population. This was further examined by mapping QTL for the two densities separately. Of the eight QTL that were detected for FT at a planting density of 16,500 plants ha^−1^, only three coincided with QTL identified at a planting density of 49,000 plants ha^−1^. Of the regions that were found to overlap between the IBM RIL and Syn14 populations, those on chromosomes 1 and 8 were identified at both planting densities, whereas the region on chromosome 2 was identified for the lower planting density only (Figure 2A). Likewise, one region that overlapped between populations for PH was the only QTL identified to be shared between the two planting densities (Figure 3A). Although this region was the most significant region identified for linkage mapping at both densities, it was not the most significant region for the IBM Syn14 population. There was also an additional overlap between the Syn14 and RIL QTL identified in the higher density treatment that was not identified in the lower-density treatment (Figure 3A). These results suggest that either the overlapping regions between the two populations or the BSA sequencing method are density-independent.

Due to the correlation between FT and PH (Irish and Nelson 1991), regions that were significant for both traits in the two populations were identified. Overlaps between regions identified for the two traits existed on chromosomes 4 (205–226 Mb; PH in Syn14 and FT in high density RIL), 5 (176–186 Mb; PH and FT in Syn14), 6 (138–147 Mb; PH and FT in Syn14), and 9 (4–6 Mb; FT in low-density RIL and PH in high-density RIL). In each of these instances, when B73 conferred the early allele, Mo17 conferred the tall allele, or vice versa. This is consistent with the expectation that when overlap between regions identified for the two traits was present, their parental contributions would be in opposite directions (*i.e.*, short plants would flower earlier).

A comprehensive list of 149 *a priori* candidate genes associated with FT was previously compiled for comparison to homologous sequences in maize (Chen *et al.* 2012). This list was used to search for BSA sequencing QTL overlapping FT candidate genes. Three putative FT genes fell within a 18.9-Mb region on chromosome 8, one of which, GRMZM2G700665, *ZmRap2.7*, and its regulatory element (*Vgt1*) 70 kb upstream have been previously shown to be associated with FT in maize (see Figure S1H) (Salvi *et al.* 2007). The maize homolog for *EMF1* (Aubert *et al.* 2001) and *ZmRap2.7* both fell within the overlapping region between the two mapping methods. A third gene GRMZM2G363429, a homolog of *BR6ox2* (Shimada *et al.* 2003), while not contained within the overlapping region between the two mapping methods, was still contained in a region identified using the BSA sequencing method on chromosome 8. In total, 9 of the 14 regions identified either contained or were within 5 Mb of putative candidate flowering time genes (see Figure S1).

Regions identified for PH were compared to physical locations of known maize dwarf mutants and genes annotated for involvement with synthesis, transport, metabolism, and signaling of gibberellins and brassinosteroids. Both of these signaling pathways have been shown to have an impact on plant height (Fernandez *et al.* 2009). Of the regions identified for PH, only one region coincided with a candidate gene, whereas two others fell within 5 Mb of putative candidate PH genes. GRMZM2G017606, a maize homolog of *shi* (Fridborg *et al.* 1999), fell within the 21.2 Mb region identified in the Syn14 population on chromosome 4 (see Figure S2).

Additionally, a recent publication (Peiffer *et al.* 2014) identified PH-associated QTL and quantitative trait nucleotides (QTN) using joint-linkage QTL mapping and a joint-linkage-assisted genome-wide association study (GWAS) within the maize US-NAM population, along with GWAS in the maize North Central Region Plant Introduction Station (NCRPIS) diversity panel. The NAM population shares its reference line B73 (McMullen *et al.* 2009) with the two populations used in this study. The two most significant regions identified for PH using the BSA sequencing method, on chromosomes 4 and 6, coincided with regions identified in the maize NAM population. The BSA sequencing region on chromosome 4 (205.2–226.3 Mb) encompassed a QTN identified by joint-linkage-assisted GWAS, whereas the region on chromosome 6 (138.2–147.9 Mb) encompassed two QTL identified using joint-linkage QTL mapping in the maize NAM population (Peiffer *et al.* 2014). Finally, the BSA sequencing region that overlapped with linkage mapping on chromosome 9 (100.9–103.2 Mb) fell within approximately 2.4 Mb from a joint-linkage QTL in the maize NAM population. For the NAM QTN identified on chromosome 4, B73 conferred the tall allele, whereas in the Syn14 population Mo17 was conferring the tall allele. This is consistent with the nearby NAM QTL (235 Mb) in which Mo17 is also conferring the tall allele. Conversely, whereas the two NAM QTL located on chromosome 6 were in repulsion phase with one another, their cumulative additive effect was in favor of Mo17 conferring the tall allele. Both this region and that on chromosome 9 are consistent with the findings in the IBM Syn14 population.

Likewise, of the regions identified for FT with BSA sequencing, three coincided with regions identified using joint-linkage QTL mapping in the maize US-NAM population. FT-associated NAM markers located on chromosomes 1 and 8 (Buckler *et al.* 2009) were encompassed by regions identified in the Syn14 population. Finally, the 3-Mb region identified on chromosome 10 fell within approximately 500 Kb of an FT-associated NAM marker (Buckler *et al.* 2009). These colocalizations of QTL, for both of the traits examined in this study, further demonstrate the ability of using the BSA sequencing method in the context of a heterogeneous population such as the IBM Syn14 to dissect quantitative traits in maize.

The feasibility of BSA on whole genome sequencing has already been described in similar studies using model organisms such as yeast (Ehrenreich *et al.* 2010; Magwene *et al.* 2011). Additionally, this approach has also been recently used in other important agronomic systems such as rice (Takagi *et al.* 2013). All of these studies have demonstrated the ability for the rapid detection of QTL from next-generation sequencing on pooled samples. Methods that use BSA with other next-generation sequencing technologies such as RNAseq have also demonstrated the ability to map genes contributing to quantitative traits in agronomic crops such as maize and wheat (Liu *et al.* 2012; Trick *et al.* 2012). However, some limitations may still exist for these types of methods.

For instance, plant height in maize demonstrates a considerable amount of heterosis (Uzarowska *et al.* 2007). Additionally, within this experiment we observed a shift of the IBM Syn14 population toward taller individuals relative to the IBM RILs, suggesting a potential mode of dominance. Through simulations, it has been shown that BSA with whole genome sequencing methods have difficulty detecting weaker effect QTL demonstrating levels of dominance (Takagi *et al.* 2013). In extreme situations of overdominance, where phenotypic effects between two homozygous states are indistinguishable relative to the phenotypic effect of the heterozygous state, it is expected that such QTL would become undetectable, as would also be the case with traditional linkage mapping in inbred populations. In such cases, it would be beneficial to have genomic information on all selected individuals rather than a pooled sample, and thus heterozygous states would be distinguishable from homozygous states. In this study, it is possible that all detected sites had lower to no dominance effects, and with additional information on all selected individuals, more QTL contributing to PH and FT could have been detected. Therefore, for traits that have higher levels of dominance like plant height in maize, a single pooling method may not be as applicable.

Typical QTL mapping studies rely on crosses of two or a small number of lines, limiting the alleles that are sampled. Because the BSA sequencing method relies only on differences in allele frequency, it is expected that this method could be applied to different structured populations of maize, especially those that do not rely on a biparental lineage. Sequencing of pooled DNA samples has also been used to scan for genomic sweeps generated through the process of artificial selection for increased ear number, as well as seed size in maize populations with multiple founder lines (Beissinger *et al.* 2014; Hirsch *et al.* 2014). Using a modified F_st_ calculated between estimated allele frequencies from sequenced samples of pooled individuals from the latest cycle of selection compared to the pool of individuals from the original population, 28 genomic regions were found to be affected by the selection for ear number (Beissinger *et al.* 2014). When this same approach was applied to a population divergently selected for 30 generations for seed size, 23 regions were identified to be causative for seed size when comparing the large and small selected populations (Hirsch *et al.* 2014). When scanning for unidirectional selection from cycle 0 for both larger or smaller seed size, 63 and 27 regions were identified, respectively (Hirsch *et al.* 2014). In this context, the BSA sequencing method could potentially be thought of as a single-generation selection experiment. Although gametes were not allowed to recombine after each cycle of selection, allele frequencies are divergently driven in the two selected pools.

This study demonstrates the feasibility of using a BSA sequencing approach to rapidly identify QTL for two traits important for the production of lignocellulosic ethanol in maize while also yielding a context for selection on genomic regions using a higher selection pressure concurrently with a large population size. Although the regions identified were too large for single gene identification, these could still potentially be used for molecular breeding efforts.

## 

## Supplementary Material

Supporting Information
