# Supplementary material for: Shared Genomic Regions Between Derivatives of a Large Segregating Population of Maize Identified Using Bulked Segregant Analysis Sequencing and Traditional Linkage Analysis
Source: G3 (Bethesda). 2015 Jun 1;5(8):1593–602. doi: 10.1534/g3.115.017665 (PMC4528316; doi:10.1534/g3.115.017665)
Supplement: Supporting Information [file supp_5_8_1593__index.html]

Shared Genomic Regions Between Derivatives of a Large Segregating Population of Maize Identified Using Bulked Segregant Analysis Sequencing and Traditional Linkage Analysis — Supporting Information 

# Shared Genomic Regions Between Derivatives of a Large Segregating Population of Maize Identified Using Bulked Segregant Analysis Sequencing and Traditional Linkage Analysis

## Supporting Information for Haase *et al.*, 2015

**Files in this Data Supplement:**

- Supporting Information - Tables S1-S3, File S1, and Figures S1-S2 (PDF, 400 KB)
- Table S1 - List of Intermated B73 X Mo17 (IBM) recombinant inbred lines (RILs) and corresponding low density phenotypes. (PDF, 127 KB)
- Table S2 - List of Intermated B73 X Mo17 (IBM) recombinant inbred lines (RILs) and corresponding high density phenotypes. (PDF, 127 KB)
- Table S3 - List of Selected Intermated B73 X Mo17 (IBM) Syn14 plants and corresponding phenotypes. (PDF, 117 KB)
- File S1 - Supplemental Method: Annotated R code. (PDF, 134 KB)
- Figure S1 - Flowering time candidate gene overlap. (PDF, 863 KB)
- Figure S2 - Plant height candidate gene overlap. (PDF, 394 KB)
