# Supplementary material for: Shared Genomic Regions Between Derivatives of a Large Segregating Population of Maize Identified Using Bulked Segregant Analysis Sequencing and Traditional Linkage Analysis
Source: G3 (Bethesda). 2015 Jun 1;5(8):1593–602. doi: 10.1534/g3.115.017665 (PMC4528316; doi:10.1534/g3.115.017665)
Supplement: Supporting Information [file supp_g3.115.017665_017665SI.pdf]

G3 · Genes | Genomes | Genetics

Genetic Dissection of Quantitative Traits Using a Bulked Segregant Analysis (BSA)-Sequencing Method on a Large Segregating Population of Maize

Nicholas Haase[*], Timothy Beissinger[§], Candice N. Hirsch[†], Brieanne Vaillancourt[‡,**], Shweta Deshpande[§§], Kerrie Barry[§§], C. Robin Buell[‡,**], Shawn M. Kaeppler[*,††], Natalia de Leon[*,††,1]

*Department of Agronomy, University of Wisconsin-Madison, Madison, WI 53706, §Department of Plant Sciences, University of California Davis, Davis, CA 95616, †Department of Agronomy and Plant Genetics, University of Minnesota, Saint Paul, MN 55108, ‡Department of Plant Biology, Michigan State University, East Lansing, MI 48824, **Department of Energy Great Lakes Bioenergy Research Center, Michigan State University, East Lansing, MI 48824, §§Department of Energy, Joint Genome Institute, Walnut Creek, CA 94598, ††Department of Energy Great Lakes Bioenergy Research Center, University of Wisconsin-Madison, Madison, WI 53706

[1] Corresponding Author: University of Wisconsin-Madison, Department of Agronomy, Madison, WI 53706. E-mail: ndeleongatti@wisc.edu

DOI: 10.1534/g3.115.017665

Table S1 List of Intermated B73 X Mo17 (IBM) recombinant inbred lines (RILs) and corresponding low density phenotypes. List of IBM RIL genotypes included in the density experiment along with plant height (PH) collected on five individual plants (P1-P5) per plot and flowering time (growing degree days [GDD]) per plot for each RIL when evaluated at a density of 16,500 plants ha^{-1} using two field replications (R1 and R2).

Geno	R1PH1	R1PH2	R1PH3	R1PH4	R1PH5	R1GDD	R2PH1	R2PH2	R2PH3	R2PH4	R2PH5	R2GDD
M0001	150	.	170	180	145	937	.	.	130	135	.	937
M0005	.	180	175	170	180	905	180	185	185	185	185	964
M0007	160	150	165	165	165	825	145	150	150	155	145	825
M0008	.	155	130	155	155	1013	.	160	.	170	150	1040
M0009	170	110	160	165	.	905	190	.	.	.	175	964
M0010	.	.	.	195	180	937	.	185	205	195	190	937
M0012	140	165	145	135	150	876	145	140	.	.	145	825
M0014	.	140	180	150	150	850	.	140	145	150	155	905
M0016	.	75	120	120	120	799	140	140	140	140	140	825
M0017	175	170	185	175	175	876	180	.	170	165	170	825
M0018	.	125	150	130	120	876	125	130	.	115	.	876
M0019	.	160	170	130	165	964	.	165	150	150	.	1013
M0021	160	165	155	165	160	850	155	155	170	.	.	850
M0022	145	.	.	145	165	964	145	145	160	140	.	905
M0023	.	145	140	155	155	905	.	.	125	135	.	850
M0024	200	210	.	200	200	937	195	215	205	200	205	937
M0025	145	140	135	145	145	825	140	155	150	.	.	825
M0026	165	150	170	160	155	905	170	170	165	150	.	905
M0028	150	155	150	155	150	876	160	.	145	.	165	905
M0029	145	140	135	130	145	876	.	160	135	145	165	876
M0030	150	.	170	150	145	1040	.	155	150	160	160	964
M0031	180	170	200	.	160	850	.	.	.	180	190	964
M0032	190	160	165	145	170	937	.	140	185	180	155	989
M0034	130	125	130	145	135	825	125	135	.	125	125	825
M0035	100	130	135	135	.	850	.	150	145	145	130	905
M0036	.	200	180	.	.	937	180	160	180	185	170	937
M0039	180	180	.	.	195	876	195	190	180	.	190	876
M0041	175	195	.	190	.	905	180	180	150	.	150	905
M0042	.	.	155	150	135	964	140	.	150	140	145	876
M0043	120	135	130	.	130	1013	135	.	.	140	150	989
M0045	145	145	160	150	145	825	.	140	145	.	.	876
M0048	.	155	155	170	175	1084	160	.	155	165	.	1121
M0051	.	180	180	175	.	850	190	185	190	170	200	850
M0052	140	140	.	.	135	825	140	150	135	.	135	825
M0054	185	.	190	195	180	825	.	180	.	175	170	850
M0055	.	140	.	.	150	754	130	.	150	145	.	825
M0057	130	.	130	.	155	1121	145	120	120	145	135	1065
M0058	145	155	150	150	150	937	140	140	.	150	150	937
M0059	165	170	170	170	150	876	150	170	155	140	.	876
M0060	150	155	160	150	145	905	140	140	145	120	155	905
M0061	175	165	170	150	155	876	.	175	.	160	155	876
M0063	130	130	135	125	120	876	135	130	140	120	125	905
M0063	.	.	140	125	120	876	130	130	.	.	.	905
M0067	160	165	160	160	180	876	170	170	.	160	160	964
M0068	165	175	.	190	185	905	200	.	165	195	175	905
M0071	145	135	120	.	140	905	135	135	150	140	140	850
M0075	.	145	130	145	150	773	.	155	.	.	135	773
M0075	140	150	150	155	145	799	145	.	135	140	120	754
M0076	165	160	185	165	190	876	190	160	160	.	165	876
M0077	.	170	170	170	155	876	.	.	165	160	160	876
M0079	.	195	175	185	180	964	.	.	185	195	190	1013
M0080	.	165	175	155	160	850	155	160	160	155	175	876
M0081	.	180	180	190	200	964	.	170	175	185	185	964

Table S1 (cont.)

Geno	R1PH1	R1PH2	R1PH3	R1PH4	R1PH5	R1GDD	R2PH1	R2PH2	R2PH3	R2PH4	R2PH5	R2GDD
M0082	165	195	195	170	190	905	190	195	195	.	195	937
M0083	.	.	120	130	140	850	.	.	125	120	130	773
M0085	170	165	165	150	165	876	.	165	160	140	165	905
M0086	.	165	160	140	180	1084	165	160	170	160	160	937
M0087	150	.	.	150	.	825	180	.	165	.	145	773
M0090	140	140	145	155	150	937	150	150	.	.	130	905
M0092	150	135	140	120	140	773	.	135	120	.	125	754
M0097	160	145	155	170	155	799	.	150	150	.	170	825
M0098	.	140	140	140	135	728	150	135	140	130	130	773
M0099	205	.	210	200	215	1065	.	.	170	.	180	1065
M0101	175	170	155	170	135	964	.	150	.	170	140	989
M0105	125	135	130	135	130	905	.	135	130	150	115	905
M0105	.	.	130	135	140	850	.	.	125	.	130	825
M0106	.	190	185	185	.	905	195	200	190	190	190	905
M0109	.	.	.	165	160	876	170	.	170	185	170	937
M0111	160	165	.	165	.	905	.	.	185	180	205	1040
M0114	145	145	135	140	135	825	.	145	140	.	.	825
M0116	.	190	170	205	175	937	170	190	185	.	170	989
M0118	170	170	165	175	160	876	.	165	170	160	165	937
M0119	170	180	175	160	140	876	.	190	190	170	.	876
M0120	155	165	165	150	155	1065	170	85	100	110	120	964
M0121	150	150	160	170	160	937	160	155	175	160	175	989
M0123	125	130	130	135	140	850	120	130	135	140	135	905
M0124	140	135	140	130	200	799	150	150	150	.	.	964
M0125	175	180	155	175	165	905	.	155	.	.	155	937
M0126	185	160	170	160	170	876	175	165	170	145	.	850
M0127	165	170	140	.	150	850	140	.	155	160	160	937
M0128	155	180	180	185	195	964	.	190	170	160	180	937
M0128	175	.	190	160	180	905	190	190	165	175	.	937
M0129	180	135	160	175	145	937	.	.	125	125	.	905
M0131	.	155	125	140	160	850	160	150	.	140	160	850
M0132	140	130	135	135	135	773	130	.	145	140	135	773
M0133	100	130	145	125	120	773	.	155	120	150	130	876
M0134	195	170	.	165	195	964	190	150	.	180	160	964
M0138	145	115	140	145	135	799	165	165	.	150	165	876
M0141	.	190	180	205	.	1084	190	195	205	.	190	1084
M0142	110	115	.	.	.	905	.	.	115	130	145	825
M0143	155	170	.	155	.	937	.	170	155	150	.	964
M0145	145	155	.	.	175	905	.	.	140	160	.	876
M0146	175	155	165	170	160	876	.	165	160	160	.	850
M0147	175	.	160	170	160	850	190	175	170	155	165	876
M0150	150	140	145	140	.	850	.	.	.	135	150	850
M0151	160	160	165	165	.	964	.	160	155	.	.	989
M0153	135	125	125	.	150	825	.	145	155	145	140	850
M0154	.	175	170	170	155	876	180	170	.	175	175	799
M0156	140	145	145	150	140	825	160	150	160	155	150	825
M0159	160	160	.	.	160	1065	.	160	165	155	155	1013
M0160	190	185	.	190	145	1013	190	.	180	190	195	989
M0161	.	215	220	165	215	989	200	.	215	205	205	1065
M0161	215	.	220	210	210	989	210	210	215	185	210	1013
M0162	205	.	200	210	210	989	180	205	200	205	200	1013
M0163	.	145	150	150	150	876	.	145	160	185	150	876
M0164	.	140	150	160	160	1040	165	170	.	150	170	1040
M0165	140	170	160	150	150	825	155	165	140	150	.	825
M0167	150	160	.	.	.	876	170	145	150	140	140	825
M0168	150	140	150	.	.	964	150	150	140	150	155	905

Table S1 (cont.)

Geno	R1PH1	R1PH2	R1PH3	R1PH4	R1PH5	R1GDD	R2PH1	R2PH2	R2PH3	R2PH4	R2PH5	R2GDD
M0169	140	150	160	155	.	825	175	150	145	160	125	825
M0171	155	150	150	170	150	825	160	185	185	160	165	876
M0172	145	160	.	150	.	876	.	.	160	165	150	905
M0174	170	185	190	180	170	905	.	.	165	185	175	937
M0174	.	190	210	200	190	937	170	180	185	180	175	937
M0176	140	140	150	165	150	850	150	140	140	.	155	905
M0177	185	175	180	180	180	964	190	.	.	.	180	964
M0178	.	125	125	150	.	876	.	125	140	125	145	937
M0180	160	.	155	135	125	876	175	170	160	170	165	850
M0181	.	.	170	145	160	964	165	.	.	.	180	937
M0182	190	.	185	165	160	825	190	185	180	.	170	850
M0185	165	175	175	175	170	964	170	155	160	160	185	937
M0186	.	170	165	170	160	850	170	175	150	170	170	876
M0187	135	.	130	130	135	876	140	.	.	135	145	876
M0189	175	180	180	165	190	937	.	180	165	185	175	876
M0190	155	180	165	165	160	876	170	155	160	165	150	825
M0191	150	165	170	165	145	905	.	140	180	140	190	937
M0192	150	.	.	175	160	1040	140	140	.	150	.	937
M0194	150	145	130	140	140	937	130	135	140	155	145	937
M0195	.	165	175	180	160	937	160	.	.	.	175	989
M0196	.	.	.	170	160	773	175	190	180	180	185	825
M0197	130	90	.	95	120	989	125	95	95	125	.	964
M0198	175	185	195	180	165	876	185	.	190	185	220	905
M0198	.	185	185	205	190	876	190	200	180	180	195	905
M0199	.	170	170	155	145	905	150	160	.	.	.	989
M0200	150	145	145	150	160	937	145	160	150	140	.	937
M0202	135	145	160	.	135	799	145	155	150	150	145	799
M0202	155	145	150	160	130	799	.	140	140	.	.	799
M0205	150	140	140	150	140	825	.	140	130	120	.	850
M0206	.	.	125	140	.	876	150	145	140	140	150	773
M0208	210	210	210	210	.	1013	.	200	175	.	195	1013
M0210	160	160	165	160	160	876	170	180	160	160	.	905
M0214	135	130	150	155	145	989	145	140	140	.	150	905
M0215	190	170	175	185	.	964	180	185	.	185	.	1040
M0216	160	175	170	160	160	876	175	.	175	180	180	876
M0218	200	200	190	200	.	937	.	.	165	190	.	1065
M0219	170	155	180	185	.	876	180	165	170	185	170	850
M0222	130	125	135	135	130	825	140	130	140	120	130	850
M0223	160	160	150	150	150	876	135	155	175	.	205	905
M0224	115	145	140	155	135	799	155	135	.	.	150	876
M0228	145	.	140	145	.	1105	.	.	140	145	140	905
M0229	150	145	140	135	140	905	.	150	180	130	155	905
M0230	160	145	155	.	145	876	.	.	.	150	165	905
M0232	220	190	200	185	165	964	.	.	195	155	160	1013
M0233	160	145	165	180	165	876	.	180	175	175	.	905
M0236	.	140	.	.	110	964	145	135	.	150	155	905
M0236	145	150	.	150	140	905	.	160	150	135	.	964
M0237	190	225	200	.	.	964	.	195	205	205	210	989
M0238	170	170	170	175	190	989	.	175	180	.	185	1084
M0240	160	140	160	165	160	799	.	150	190	155	150	825
M0241	180	155	170	160	.	905	180	175	165	150	160	825
M0244	125	120	130	125	140	825	.	145	125	120	120	876
M0246	175	170	185	200	190	850	195	.	170	195	190	989
M0250	.	.	180	205	185	964	185	155	175	200	160	964
M0253	145	.	.	150	165	964	150	140	145	.	145	825
M0255	135	105	140	120	140	825	130	130	140	130	125	850

Table S1 (cont.)

Geno	R1PH1	R1PH2	R1PH3	R1PH4	R1PH5	R1GDD	R2PH1	R2PH2	R2PH3	R2PH4	R2PH5	R2GDD
M0255	.	.	130	120	130	825	.	.	140	125	.	850
M0256	180	185	185	185	180	937	185	.	175	.	190	964
M0258	140	150	130	140	130	876	130	135	140	135	130	850
M0261	190	.	.	180	.	1105	180	175	190	.	190	1013
M0262	150	160	145	155	.	905	155	140	155	.	150	876
M0263	125	145	140	145	145	850	.	140	135	140	150	825
M0264	155	120	110	120	100	905	115	130	120	120	110	937
M0265	140	140	140	140	140	773	135	145	140	.	125	825
M0266	165	180	150	145	140	773	.	140	145	140	150	876
M0267	.	.	.	120	140	937	.	155	.	155	115	937
M0268	145	140	140	165	140	905	.	160	150	140	150	876
M0269	130	135	140	100	130	876	110	120	125	135	140	825
M0270	170	175	175	180	195	876	185	170	175	170	185	876
M0271	160	145	160	170	180	876	150	140	150	.	145	905
M0274	.	.	135	160	160	825	160	155	.	.	150	937
M0275	165	170	160	130	.	964	135	.	.	145	150	964
M0276	140	155	150	145	160	1013	175	150	145	140	140	964
M0277	165	140	135	145	145	825	130	.	135	.	130	773
M0279	170	175	175	170	.	1040	.	170	195	170	165	1084
M0280	.	.	180	160	195	937	.	165	.	160	155	876
M0281	.	.	185	195	200	1121	185	210	190	205	215	1065
M0282	170	160	165	160	170	850	160	160	170	170	160	850
M0283	160	145	180	175	165	825	160	175	165	170	170	825
M0284	170	180	.	170	195	799	120	160	.	175	.	905
M0287	.	195	205	210	210	989	.	.	210	190	.	964
M0288	165	.	.	.	160	850	.	155	.	.	145	773
M0289	.	.	170	170	180	754	.	180	175	195	170	825
M0295	150	160	160	.	.	905	155	160	.	140	145	876
M0296	170	150	175	.	170	1013	160	140	180	150	155	1040
M0297	150	145	.	145	155	876	150	170	135	.	130	876
M0298	135	135	135	160	.	876	140	155	.	.	160	964
M0300	215	195	175	185	195	964	.	190	195	200	.	989
M0301	.	160	160	175	135	876	175	165	150	155	180	876
M0303	.	.	.	145	.	989	.	.	.	.	.	1084
M0303	120	150	120	110	135	1013	115	.	140	105	.	1065
M0304	.	140	155	155	165	964	165	170	140	.	170	964
M0305	140	140	135	120	150	825	150	.	150	.	115	876
M0307	.	.	145	145	145	825	.	135	140	140	110	876
M0308	160	160	190	170	195	989	.	190	180	155	195	989
M0309	160	165	180	155	.	850	165	.	170	160	.	825
M0310	125	150	150	130	150	937	130	155	125	150	140	905
M0311	135	135	135	140	140	799	140	145	120	140	140	773
M0313	.	160	150	150	135	905	.	145	.	145	135	876
M0314	170	150	.	160	175	1013	170	165	165	.	.	1013
M0317	155	160	160	135	155	964	155	160	120	140	160	964
M0318	.	175	160	190	175	850	170	.	165	150	.	876
M0318	185	180	185	195	180	825	160	180	165	160	175	850
M0321	.	.	.	170	160	964	155	.	175	175	175	964
M0322	170	.	180	165	170	964	.	.	200	200	.	1121
M0323	170	.	175	170	165	850	.	170	185	190	160	905
M0325	210	200	175	200	180	850	.	180	210	185	220	773
M0328	215	195	205	220	.	937	210	.	.	220	210	989
M0329	.	.	150	170	150	905	.	.	160	140	155	876
M0331	180	185	.	175	.	850	190	185	185	195	.	905
M0332	155	155	155	145	145	964	.	160	160	.	.	964
M0334	130	140	110	125	120	825	105	110	.	110	120	754

Table S1 (cont.)

Geno	R1PH1	R1PH2	R1PH3	R1PH4	R1PH5	R1GDD	R2PH1	R2PH2	R2PH3	R2PH4	R2PH5	R2GDD
M0335	180	.	.	160	155	1013	.	160	.	135	.	964
M0335	.	.	160	150	180	937	.	.	170	145	145	1065
M0336	.	180	140	150	150	754	160	155	155	170	155	825
M0337	200	205	200	200	200	876	170	210	200	210	185	876
M0338	165	140	.	120	115	850	175	140	185	180	150	825
M0340	.	.	.	.	.	1013	175	195	145	.	165	1013
M0340	.	170	.	.	.	937	.	.	165	170	180	937
M0341	170	190	155	160	160	964	170	120	.	180	.	1040
M0342	190	180	160	155	165	989	200	210	210	180	200	1040
M0342	185	190	190	185	180	964	175	180	.	180	180	937
M0344	185	175	200	175	.	964	190	140	.	145	175	1040
M0346	.	185	185	.	200	1013	180	190	180	190	.	989
M0349	.	130	.	130	.	937	.	.	.	.	.	1121
M0349	.	.	135	120	140	964	140	130	155	160	135	989
M0351	.	.	.	.	125	1084	.	.	.	.	.	825
M0351	.	.	.	90	140	964	.	.	.	.	.	964
M0352	.	.	135	120	130	825	.	130	115	125	130	773
M0353	.	.	190	175	195	1065	185	180	185	180	185	1065
M0356	160	155	165	165	165	876	.	145	160	145	145	876
M0356	160	.	.	.	160	876	155	145	165	155	155	905
M0357	.	145	155	150	150	876	135	155	145	130	135	937
M0358	.	150	155	180	150	876	155	175	170	.	165	876
M0360	.	160	150	155	150	825	155	140	145	140	.	850
M0365	180	175	165	165	170	876	180	180	175	170	155	876
M0368	170	160	155	160	195	825	.	185	170	170	180	989

Table S2 List of Intermated B73 X Mo17 (IBM) recombinant inbred lines (RILs) and corresponding high density phenotypes. List of IBM RIL genotypes included in the density experiment along with plant height (PH) collected on five individual plants (P1-P5) per plot and flowering time (growing degree days [GDD]) per plot for each RIL when evaluated at a density of 49,000 plants ha^{-1} density using two field replications (R1 and R2).

Geno	R1PH1	R1PH2	R1PH3	R1PH4	R1PH5	R1GDD	R2PH1	R2PH2	R2PH3	R2PH4	R2PH5	R2GDD
M0001	185	175	185	190	175	1467	140	140	135	150	150	1467
M0005	.	220	220	235	215	1467	220	210	220	215	200	1435
M0007	160	180	180	180	175	1355	.	.	180	155	155	1355
M0008	195	195	200	180	195	1467	180	180	185	180	160	1519
M0009	160	200	220	170	165	1406	180	180	180	185	170	1467
M0010	230	210	200	210	210	1435	215	215	225	220	220	1467
M0012	175	175	180	180	175	1355	165	165	165	155	145	1406
M0014	175	180	180	170	170	1329	155	160	160	165	180	1355
M0016	135	150	160	160	150	1284	.	.	.	.	.	1258
M0017	190	190	190	200	190	1303	185	180	190	200	185	1303
M0018	.	.	.	.	.	.	145	145	165	150	150	1355
M0019	190	200	185	180	170	1435	210	185	190	185	180	1570
M0021	200	190	185	200	170	1284	190	190	190	200	180	1355
M0022	175	190	190	195	175	1467	190	185	175	180	165	1494
M0023	170	160	160	160	170	1406	170	155	155	160	155	1406
M0024	225	230	230	230	230	1435	225	215	230	215	205	1435
M0025	170	170	180	180	175	1284	170	165	155	160	160	1284
M0026	190	195	175	185	175	1406	195	185	180	170	170	1406
M0028	175	180	170	175	180	1380	175	185	190	185	180	1355
M0029	160	165	175	165	175	1406	180	155	160	170	155	1406
M0030	190	190	195	195	200	1380	165	170	185	180	190	1435
M0031	220	220	220	210	220	1329	205	190	200	185	185	1406
M0032	205	205	210	220	220	1406	195	195	205	190	190	1467
M0034	150	165	160	165	170	1303	160	150	170	170	155	1380
M0035	145	140	165	135	145	1406	145	165	145	160	160	1380
M0036	210	195	195	200	200	1406	205	180	195	175	200	1406
M0039	210	215	210	205	215	1380	205	205	205	200	200	1406
M0041	205	215	215	215	205	1406	205	195	210	195	210	1435
M0042	160	160	165	155	160	1355	160	160	165	160	160	1406
M0043	185	180	185	185	185	1355	190	175	170	170	175	1303
M0045	160	175	175	170	185	1355	145	175	180	160	165	1406
M0048	175	155	150	165	175	1519	175	185	185	175	175	1494
M0051	205	210	195	205	220	1355	220	195	205	205	210	1355
M0052	165	170	170	160	180	1303	150	160	165	175	160	1303
M0054	220	225	210	230	220	1303	200	200	210	210	200	1406
M0055	170	160	155	170	155	1284	155	150	155	150	145	1355
M0057	180	135	125	140	125	1543	155	160	195	165	180	1570
M0058	170	165	190	160	170	1435	170	190	195	160	185	1543
M0059	210	200	205	200	210	1406	185	180	195	195	180	1406
M0060	170	170	165	160	165	1355	175	180	175	180	160	1435
M0061	175	165	180	190	165	1303	180	195	185	170	200	1380
M0063	.	155	140	145	150	1329	160	160	150	150	145	1329
M0063	145	145	135	135	135	1329	165	145	150	145	135	1406
M0067	190	190	200	170	170	1406	180	175	180	170	185	1406
M0068	220	230	240	220	260	1406	220	195	220	185	200	1467
M0071	175	170	160	160	170	1329	165	150	175	165	155	1355
M0075	.	150	175	170	160	1258	160	175	170	165	165	1258
M0075	215	175	165	180	155	1284	160	165	155	165	160	1258
M0076	195	195	185	205	205	1406	215	205	215	210	215	1435
M0077	210	210	205	200	210	1467	180	185	190	180	175	1406
M0079	200	200	200	190	185	1467	205	175	185	195	180	1467
M0080	175	180	175	170	180	1355	155	150	160	165	155	1380
M0081	205	195	210	205	205	1406	195	200	190	190	205	1406

Table S2 (cont.)

Geno	R1PH1	R1PH2	R1PH3	R1PH4	R1PH5	R1GDD	R2PH1	R2PH2	R2PH3	R2PH4	R2PH5	R2GDD
M0082	210	240	240	220	220	1435	215	225	220	210	190	1614
M0083	140	145	140	155	155	1258	140	130	145	155	145	1303
M0085	205	190	200	205	185	1380	180	190	200	200	205	1406
M0086	195	190	190	195	195	1406	195	200	190	200	200	1435
M0087	190	170	180	190	180	1258	180	170	170	175	165	1284
M0090	150	175	160	165	175	1467	150	150	160	160	145	1494
M0092	160	150	145	140	160	1258	175	160	140	145	150	1258
M0097	170	175	180	170	175	1355	175	155	160	160	165	1284
M0098	180	180	185	185	180	1284	170	135	145	160	145	1258
M0099	215	235	215	240	220	1519	230	235	230	230	240	1635
M0101	200	185	200	160	180	1329	190	160	165	160	175	1303
M0105	160	165	160	160	155	1329	170	170	165	150	160	1355
M0105	150	150	165	175	165	1355	145	160	160	175	155	1380
M0106	220	220	230	220	230	1380	225	185	195	200	190	1435
M0109	180	165	180	220	195	1467	200	200	200	220	200	1406
M0111	205	210	180	205	195	1406	185	200	200	190	185	1406
M0114	155	160	170	175	165	1303	165	175	170	165	155	1303
M0116	220	210	205	220	225	1406	205	205	200	210	215	1467
M0118	195	185	185	180	185	1406	190	185	180	195	190	1467
M0119	185	170	185	185	180	1380	.	.	.	190	190	1380
M0120	195	180	190	190	170	1494	160	180	170	180	185	1494
M0121	180	175	170	165	175	1406	190	195	190	185	190	1467
M0123	170	175	160	170	165	1329	155	150	155	150	150	1406
M0124	170	175	170	175	170	1355	155	145	160	150	140	1406
M0125	185	190	190	185	190	1303	185	180	190	195	185	1406
M0126	220	210	200	190	205	1406	180	180	195	180	175	1406
M0127	185	160	180	180	170	1355	180	185	170	165	170	1435
M0128	.	190	200	175	180	1467	195	210	195	195	185	1467
M0128	225	200	190	225	195	1467	185	195	200	215	210	1519
M0129	165	165	180	195	185	1467	190	175	215	180	165	1435
M0131	180	175	155	155	175	1284	160	145	160	165	150	1380
M0132	170	160	165	165	165	1284	170	180	170	165	170	1258
M0133	165	165	165	150	160	1303	160	175	180	160	165	1303
M0134	180	190	210	190	195	1435	190	190	175	185	190	1467
M0138	165	155	155	155	180	1284	165	160	160	165	160	1355
M0141	220	205	195	205	220	1519	205	200	215	210	220	1519
M0142	160	160	155	160	170	1435	150	140	150	140	145	1284
M0143	185	170	150	180	185	1467	170	175	180	160	170	1467
M0145	190	180	170	170	205	1329	165	165	175	175	180	1406
M0146	195	190	205	190	195	1355	190	185	170	180	195	1355
M0147	210	210	195	210	210	1380	190	190	190	180	190	1435
M0150	160	160	165	160	160	1355	165	180	175	170	175	1355
M0151	190	160	190	185	180	1435	160	175	175	180	165	1494
M0153	170	175	175	160	170	1380	170	165	155	170	175	1406
M0154	210	200	200	200	200	1284	200	210	195	195	190	1303
M0156	165	170	175	165	170	1303	175	170	170	180	175	1303
M0159	150	195	170	195	170	1595	160	155	185	165	155	1543
M0160	220	215	220	190	205	1467	200	210	210	190	205	1467
M0161	230	225	230	230	225	1494	235	240	235	240	235	1543
M0161	240	215	240	235	235	1519	220	225	220	225	225	1570
M0162	235	210	230	215	235	1467	220	230	215	225	225	1543
M0163	190	190	185	180	200	1406	185	185	190	190	190	1406
M0164	205	190	185	200	185	1519	195	170	205	205	200	1543
M0165	160	170	170	180	155	1303	180	175	175	170	160	1355
M0167	160	165	160	165	165	1303	165	160	160	160	160	1303
M0168	190	190	195	180	175	1435	170	155	175	160	175	1435

Table S2 (cont.)

Geno	R1PH1	R1PH2	R1PH3	R1PH4	R1PH5	R1GDD	R2PH1	R2PH2	R2PH3	R2PH4	R2PH5	R2GDD
M0169	170	180	175	180	175	1303	180	185	190	190	170	1355
M0171	200	200	200	195	195	1380	185	165	180	205	180	1406
M0172	160	185	185	180	185	1355	185	185	185	175	170	1406
M0174	225	220	230	220	220	1406	205	210	215	220	210	1406
M0174	205	210	210	210	210	1467	195	210	210	200	210	1467
M0176	165	185	190	180	190	1355	170	190	180	155	160	1406
M0177	195	190	180	190	195	1406	210	215	205	200	195	1467
M0178	175	180	170	165	170	1329	150	155	145	135	140	1380
M0180	160	180	170	160	165	1355	180	170	170	180	175	1355
M0181	190	200	200	210	195	1406	180	170	185	190	185	1467
M0182	190	200	200	210	180	1329	205	195	210	205	210	1380
M0185	185	190	195	180	180	1435	210	200	210	215	200	1435
M0186	200	210	200	200	205	1355	195	200	170	180	190	1406
M0187	155	160	170	160	170	1355	155	155	140	150	135	1355
M0189	190	190	195	195	195	1406	190	180	185	175	205	1435
M0190	190	.	185	175	190	1355	190	195	190	190	205	1406
M0191	170	175	165	150	160	1355	190	180	180	180	210	1435
M0192	165	150	170	175	165	1467	175	175	180	180	180	1467
M0194	150	150	165	150	155	1355	145	145	145	160	150	1406
M0195	190	200	195	180	200	1467	200	200	200	200	210	1519
M0196	210	210	240	205	205	1284	210	230	210	210	195	1284
M0197	.	.	.	.	.	1467	115	155	145	115	115	1467
M0198	230	215	220	215	215	1355	205	210	210	215	210	1380
M0198	230	230	230	225	235	1406	210	210	210	210	200	1406
M0199	195	195	195	190	195	1467	185	180	195	175	170	1406
M0200	160	185	170	170	185	1435	160	165	185	185	175	1435
M0202	175	175	150	185	180	1258	170	160	165	165	165	1284
M0202	180	190	175	170	175	1355	150	160	155	160	150	1303
M0205	185	180	185	190	170	1329	160	140	140	145	165	1435
M0206	180	175	180	180	180	1258	160	170	165	155	170	1284
M0208	210	205	215	240	210	1543	215	210	240	240	235	1519
M0210	190	195	190	180	200	1355	180	185	190	190	190	1406
M0214	170	185	160	155	160	1467	175	180	180	175	165	1467
M0215	190	195	190	165	190	1467	205	185	155	180	195	1570
M0216	160	185	195	185	175	1406	175	185	180	180	175	1406
M0218	190	190	195	230	225	1467	225	220	215	220	215	1467
M0219	205	160	195	185	180	1380	175	190	200	200	215	1355
M0222	155	145	155	150	150	1355	160	150	150	165	135	1284
M0223	190	185	190	190	200	1329	170	160	165	180	180	1303
M0224	185	180	170	175	170	1303	170	170	145	165	170	1467
M0228	155	160	135	135	145	1467	150	150	140	150	145	1406
M0229	200	190	190	190	190	1380	170	175	190	175	180	1435
M0230	165	180	175	160	155	1355	170	160	160	160	155	1303
M0232	175	175	220	220	165	1494	220	215	215	230	230	1467
M0233	210	180	200	210	195	1355	195	200	200	200	190	1406
M0236	170	180	175	185	185	1380	170	160	160	165	160	1406
M0236	185	175	175	195	190	1406	155	170	160	160	150	1406
M0237	235	225	230	230	215	1467	235	240	240	200	245	1467
M0238	225	225	225	225	230	1467	205	210	210	200	190	1543
M0240	150	150	175	160	170	1284	175	165	160	180	165	1303
M0241	210	215	210	205	210	1406	160	200	200	220	180	1519
M0244	140	130	150	160	145	1355	140	145	155	130	125	1355
M0246	.	220	205	200	210	1435	200	195	190	190	190	1467
M0250	225	210	210	225	220	1435	220	220	220	225	235	1467
M0253	160	170	165	160	145	1303	160	160	175	165	170	1467

Table S2 (cont.)

Geno	R1PH1	R1PH2	R1PH3	R1PH4	R1PH5	R1GDD	R2PH1	R2PH2	R2PH3	R2PH4	R2PH5	R2GDD
M0255	160	160	160	165	150	1303	135	130	110	130	125	1284
M0255	150	130	165	160	155	1355	165	150	145	140	155	1380
M0256	200	205	210	205	210	1406	200	200	210	190	200	1519
M0258	150	155	165	155	150	1355	145	145	145	145	150	1355
M0261	200	200	195	195	195	1519	190	195	190	180	190	1570
M0262	210	200	195	185	190	1355	185	190	170	165	170	1406
M0263	160	180	180	170	165	1355	170	165	170	165	165	1355
M0264	150	155	150	145	150	1380	130	130	140	130	130	1406
M0265	155	135	150	155	170	1303	140	160	155	155	155	1406
M0266	170	175	170	160	165	1355	170	190	175	175	170	1355
M0267	165	160	165	160	170	1329	135	135	150	150	120	1303
M0268	165	165	155	160	170	1406	165	160	150	140	155	1406
M0269	170	175	170	170	160	1329	160	145	155	155	150	1380
M0270	190	190	195	200	195	1355	185	185	200	205	185	1406
M0271	205	205	195	200	195	1355	190	190	195	185	185	1494
M0274	195	195	200	185	185	1329	180	190	180	190	180	1380
M0275	175	180	170	165	155	1406	165	170	170	175	170	1406
M0276	160	160	180	170	170	1380	175	170	175	155	165	1380
M0277	175	180	190	175	180	1303	180	170	175	165	160	1284
M0279	200	215	210	195	195	1543	.	.	.	140	175	1570
M0280	220	200	225	200	190	1303	205	155	175	200	185	1467
M0281	220	220	230	.	230	1635	200	225	190	200	210	1519
M0282	180	195	180	185	190	1355	180	170	175	180	185	1380
M0283	180	185	195	190	200	1284	205	190	190	190	195	1303
M0284	195	215	200	210	200	1303	190	205	210	210	205	1329
M0287	220	225	225	225	215	1494	210	210	225	220	210	1519
M0288	175	175	165	170	170	1303	205	175	170	165	170	1284
M0289	210	135	200	210	210	1303	200	205	185	195	195	1329
M0295	180	185	180	175	190	1380	175	175	180	185	185	1406
M0296	185	180	180	170	160	1467	185	170	160	170	140	1570
M0297	180	195	180	190	175	1355	175	175	165	170	180	1406
M0298	155	160	160	175	145	1303	155	155	145	140	160	1303
M0300	225	210	220	220	200	1467	185	175	200	225	210	1595
M0301	185	175	150	190	140	1303	160	180	160	160	180	1284
M0303	.	170	145	165	145	1519	160	160	160	160	145	1519
M0303	130	160	155	155	130	1543	175	120	145	170	175	1543
M0304	180	190	185	190	200	1329	175	185	190	180	160	1435
M0305	170	165	170	165	175	1355	170	165	170	175	165	1355
M0307	155	170	180	175	170	1303	180	165	175	165	145	1406
M0308	220	220	185	200	200	1467	210	200	190	190	190	1570
M0309	180	190	190	195	190	1303	200	190	185	210	180	1284
M0310	145	145	150	145	125	1406	165	115	125	130	145	1467
M0311	185	160	165	165	160	1284	160	175	180	165	150	1355
M0313	165	165	160	160	170	1303	155	200	175	170	170	1435
M0314	180	185	175	180	185	1435	190	180	190	190	195	1519
M0317	180	205	195	155	185	1355	175	175	195	175	185	1494
M0318	215	215	220	220	220	1284	.	170	180	205	175	1355
M0318	210	205	205	205	220	1303	190	215	210	190	180	1355
M0321	150	180	170	165	170	1435	190	185	190	195	195	1467
M0322	210	220	205	220	210	1406	210	210	220	220	200	1435
M0323	190	195	155	180	180	1355	195	180	200	190	190	1355
M0325	235	230	240	230	220	1329	220	215	215	240	235	1355
M0328	230	230	230	235	235	1435	230	235	230	230	240	1467
M0329	.	185	160	160	170	1355	.	170	165	280	155	1406
M0331	210	230	220	225	190	1406	205	175	205	195	215	1435

Table S2 (cont.)

Geno	R1PH1	R1PH2	R1PH3	R1PH4	R1PH5	R1GDD	R2PH1	R2PH2	R2PH3	R2PH4	R2PH5	R2GDD
M0332	170	170	170	160	165	1329	150	155	180	160	155	1380
M0334	130	135	135	130	140	1258	130	130	135	140	135	1258
M0335	175	180	180	185	175	1329	180	180	160	170	170	1406
M0335	175	190	205	190	180	1406	190	180	200	180	180	1494
M0336	185	185	190	185	180	1284	180	175	195	190	180	1303
M0337	200	210	210	210	210	1355	195	210	215	215	235	1355
M0338	180	180	170	170	190	1258	185	185	195	175	160	1284
M0340	185	185	185	175	150	1406	190	200	195	200	195	1380
M0340	190	190	205	190	185	1406	185	195	190	210	195	1435
M0341	180	190	185	180	195	1406	165	190	190	170	195	1435
M0342	230	230	230	220	215	1467	220	215	225	220	220	1467
M0342	220	230	220	210	210	1467	225	230	220	220	220	1467
M0344	205	220	190	220	220	1467	205	200	190	200	195	1494
M0346	200	200	205	205	205	1467	.	195	210	200	190	1435
M0349	175	170	160	155	160	1435	170	160	170	190	170	1467
M0349	175	180	180	175	190	1467	165	160	175	185	185	1494
M0351	.	160	120	145	170	1303	140	130	135	155	155	1355
M0351	155	170	165	165	175	1355	150	140	160	165	155	1467
M0352	160	155	155	155	150	1258	145	140	145	150	140	1258
M0353	205	210	225	205	200	1494	190	205	195	200	205	1543
M0356	160	170	175	185	170	1355	170	170	180	175	180	1380
M0356	170	180	170	190	180	1406	170	190	165	165	160	1406
M0357	170	170	145	190	170	1355	155	205	170	175	150	1406
M0358	170	165	175	175	200	1406	180	195	180	205	210	1467
M0360	180	200	190	190	190	1303	185	185	190	180	190	1303
M0365	215	230	215	185	210	1380	200	210	210	205	205	1406
M0368	200	195	200	210	210	1355	190	190	190	180	180	1435

Table S3 List of Selected Intermated B73 X Mo17 (IBM) Syn14 plants and corresponding phenotypes. List of 92 selected extremes individuals for both plant height (PH) and flowering time (growing degree days [GDD]) from the IBM Syn14 population grown at density of 16,500 plants ha^{-1}.

Flowering Time				Plant Height			
Early Flowering Pool		Late Flowering Pool		Short Pool		Tall Pool	
Coordinate	*GDD*	*Coordinate*	*GDD*	*Coordinate*	*Plant Height(cm)*	*Coordinate*	*Plant Height(cm)*
DA_6	528	AR_51	1013	AO_47	85	R_90	260
BI_62	587	BJ_41	1013	CY_35	90	E_91	260
BL_62	587	AY _73	1013	AR_77	95	J_63	260
CH_10	587	AC_79	1040	AY_33	95	T_80	260
BI_8	617	AK_72	1040	BR_29	95	CW_68	260
BK_2	617	AM_9	1040	BT_96	105	Q_84	260
CG_20	617	AS_23	1040	U_5	105	CJ_87	260
CJ_64	617	AT_3	1040	CU_11	110	CO_88	260
CS_73	617	BC_43	1040	U_56	110	DI_80	260
DD_47	617	BE_16	1040	DJ_35	120	Q_58	260
DI_13	617	BE_29	1040	AG_65	125	CO_41	260
DL_96	617	C_15	1040	CK_24	125	DH_56	260
AT_20	645	CM_40	1040	I_11	125	M_90	260
BP_18	645	CN_12	1040	Z_39	130	DE_82	260
BU_5	645	CS_8	1040	AZ_46	135	DG_40	260
BV_1	645	CT_18	1040	BA_29	135	AK_70	265
BY_60	645	E_40	1040	BV_29	135	BL_52	265
CD_4	645	G_39	1040	CX_12	135	C_83	265
CI_3	645	J_38	1040	AB_78	140	C_87	265
CW_18	645	J_86	1040	AD_35	140	CA_51	265
CX_51	645	AH_26	1065	AT_33	140	CC_51	265
V_49	645	AJ_58	1065	AT_43	140	CI_19	265
C_13	675	AK_13	1065	AT_44	140	CI_84	265
CW_45	675	CG_16	1065	BE_24	140	CV_94	265
CU_82	675	CM_56	1065	BE_55	140	CX_59	265
CO_16	675	CO_93	1065	BI_31	140	DE_67	265
CP_14	675	CT_86	1065	BJ_78	140	DG_57	265
CO_64	675	DC_63	1065	BK_30	140	DK_56	265
CY_46	675	F_24	1065	BQ_55	140	E_88	265
BA_78	675	J_78	1065	CK_32	140	F_39	265
CF_25	675	O_48	1065	CV_41	140	J_50	265
CT_1	675	B_39	1084	DC_2	140	X_74	265
CQ_57	675	BI_41	1084	DE_49	140	A_64	270
CA_8	675	CZ_76	1084	O_40	140	BN_85	270
DB_93	675	I_25	1084	P_94	140	CB_88	270
CS_31	675	AT_44	1105	U_39	140	CC_67	270
BV_5	675	BM_31	1105	Z_72	140	CU_75	270
BY_21	675	BR_36	1105	CN_16	145	DA_68	270
DH_26	675	BT_39	1105	V_74	145	DC_55	270
CK_20	675	CC_29	1105	Y_1	145	Q_83	270
AD_36	675	DC_38	1105	CC_4	145	X_60	270
BS_10	675	Y_24	1105	DI_81	145	BW_85	275
CO_8	675	BG_38	1121	DF_48	145	Q_34	275
BD_48	675	BH_55	1121	H_18	145	AE_66	280
DJ_94	675	CK_24	1121	G_78	145	AZ_49	280
A_5	675	AF_3	1158	BP_18	145	D_80	280

File S1

Supplemental Method: Annotated R code.

Included is an annotated version of R script used for filtering SNPs, calculating Z' statistic, defining significant regions and directionality of significant regions.

```
### change working directory to read in SNP file
setwd("C:/Users/de Leon Lab/Documents")

### read in SNP file
newmbf =
read.table("B73_Mo17_extreme_allele_freq_matrix_3301371_SNPs_bias_correct_100bp_0.25_0.75_control_with_header.txt",
header=T)

### read in maize chromosome lengths file
## this is a file with bp lengths of maize chromosomes and their cumulative length
setwd("C:/Users/de Leon Lab/Documents/Single_Plant_Files/2012/Sequencing_Data/Resequencing Data")
lengths = read.table("maize_length_v2.txt",header=T)

## creating a vector of consecutive lengths
consec_pos = matrix(nrow = length(newmbf[,1]), ncol = 1)

## storage of positions
## all of the positions in matrix will be added to the cumulative length of their corresponding chromosome
consec_pos[(which(newmbf[,1] == "chr1")),] = newmbf[which(newmbf[,1] == "chr1"),2]

### loop through the remaining chromosomes
for(i in 2:10){
	chromo = paste("chr",i,sep="")
	consec_pos[(which(newmbf[,1] == chromo)),] =
	(newmbf[(which(newmbf[,1] == chromo)),2]) + (lengths[(i-1),3])
}

### attach this new vector to the existing matrix
newmbf = cbind(newmbf, consec_pos)

##########################################################################################################
### new analysis
### parse appropriate columns
## the names of each of the columns is below.
##  [1] "chr"               "pos"               "B73_allele"
##  [4] "Mo17_allele"       "control_coverage"  "control_B73_count"
##  [7] "control_Mo17_count"  "control_other_count" "control_B73_freq"
## [10] "control_Mo17_freq"   "control_other_freq"  "early_coverage"
## [13] "early_B73_count"     "early_Mo17_count"    "early_other_count"
## [16] "early_B73_freq"      "early_Mo17_freq"     "early_other_freq"
## [19] "late_coverage"       "late_B73_count"      "late_Mo17_count"
## [22] "late_other_count"    "late_B73_freq"       "late_Mo17_freq"
## [25] "late_other_freq"     "short_coverage"      "short_B73_count"
## [28] "short_Mo17_count"    "short_other_count"   "short_B73_freq"
## [31] "short_Mo17_freq"     "short_other_freq"    "tall_coverage"
## [34] "tall_B73_count"      "tall_Mo17_count"     "tall_other_count"
## [37] "tall_B73_freq"       "tall_Mo17_freq"      "tall_other_freq"
## [40] "consec_pos"

### create a subsetted matrix for each trait.
mbfFT = newmbf[,c(1:4,13:17,20:24,40)]
```

```
mbfPH = newmbf[,c(1:4,27:31,34:38,40)]

##### filter matrices by allele count, presence of other alleles, unknown chromosome, and presence of NA's
mbfFT = mbfFT[-(which((mbfFT[,5]+mbfFT[,6]) < 20)),]
mbfFT = mbfFT[-(which((mbfFT[,10]+mbfFT[,11]) < 20)),]

### remove any SNPw with > +1SD of mean
mbfFT = mbfFT[-(which((mbfFT[,5]+mbfFT[,6]) > 60.8)),]
mbfFT = mbfFT[-(which((mbfFT[,10]+mbfFT[,11]) > 60.8)),]


if(length(which(mbfFT[,7] > 0))>0) {
		mbfFT = mbfFT[-which(mbfFT[,7] > 0),]
}

if(length(which(mbfFT[,12] > 0)) > 0) {
		mbfFT = mbfFT[-which(mbfFT[,12] > 0),]
}

##### remove monomorphic SNPs
if(length(which(mbfFT[,9] == 1 & mbfFT[,14] ==1)) > 0) {
mbfFT = mbfFT[-(which(mbfFT[,9] == 1 & mbfFT[,14] == 1)),]
}
if(length(which(mbfFT[,8] == 1 & mbfFT[,13] == 1)) > 0) {
mbfFT = mbfFT[-(which(mbfFT[,8] == 1 & mbfFT[,13] == 1)),]
}

mbfFT = mbfFT[-which(mbfFT[,1] == "chrUNKNOWN"),]
mbfFT = mbfFT[-which(is.na(mbfFT[,5])),]
mbfFT = mbfFT[-which(is.na(mbfFT[,10])),]

############################################################
##### do the same with the plant height matrix

mbfPH = mbfPH[-(which((mbfPH[,5]+mbfPH[,6]) < 20)),]
mbfPH = mbfPH[-(which((mbfPH[,10]+mbfPH[,11]) < 20)),]

if(length(which(mbfPH[,7] > 0))>0) {
		mbfPH = mbfPH[-which(mbfPH[,7] > 0),]
}

if(length(which(mbfPH[,12] > 0)) > 0) {
		mbfPH = mbfPH[-which(mbfPH[,12] > 0),]
}

##### remove monomorphic SNPs

if(length(which(mbfPH[,9] == 1 & mbfPH[,14] == 1)) > 0) {
mbfPH = mbfPH[-(which(mbfPH[,9] == 1 & mbfPH[,14] == 1)),]
}
if(length(which(mbfPH[,8] ==1 & mbfPH[,13] == 1)) > 0) {
mbfPH = mbfPH[-(which(mbfPH[,8] == 1 & mbfPH[,13] == 1)),]
}

mbfPH = mbfPH[-which(mbfPH[,1] == "chrUNKNOWN"),]
mbfPH = mbfPH[-which(is.na(mbfPH[,5])),]
mbfPH = mbfPH[-which(is.na(mbfPH[,10])),]
```

```
### put the matrices in the correct order
mbfFT = mbfFT[order(mbfFT[,"consec_pos"],decreasing=F),]
mbfPH = mbfPH[order(mbfPH[,"consec_pos"],decreasing=F),]

############################################################

### calculate the statistic accross a group of 15 SNPs
library(zoo)

######### the following is the calculation of the z-stat
## z = (p_best - p_worst) / sqrt(p_hat*(1-p_hat)*((1/n1)+(1/n2)))
## p_hat = (x1 + x2) / (n1 + n2)

### create a new matrix for storing the information
ztest_ft = matrix(nrow=length(mbfFT[,1]),ncol=14)

### name the columns of the new matrix
colnames(ztest_ft) = c("Pos","B73.Freq.Diff","count1","count2","sample1",
"sample2","p_hat","Z","Avg_Z","testable_z", "P_value", "-log10(P_value)","chr","consec")

## store SNP positions
ztest_ft[,1] = mbfFT[,2]
## calculate the allele frequency difference
ztest_ft[,2] = mbfFT[,8]-mbfFT[,13]
### store the B73 allele count for each pool
ztest_ft[,3] = mbfFT[,5]
ztest_ft[,4] = mbfFT[,10]
### store total number of reads in each pool
ztest_ft[,5] = (mbfFT[,5]+mbfFT[,6])
ztest_ft[,6] = (mbfFT[,10]+mbfFT[,11])
### calculate p_hat
ztest_ft[,7] = ((ztest_ft[,"count1"]+ztest_ft[,"count2"])/(ztest_ft[,"sample1"]+ztest_ft[,"sample2"]))
### calculate the z statistic
ztest_ft[,8] = (ztest_ft[,2])/(sqrt(
        (ztest_ft[,7])*(1-(ztest_ft[,7]))*((1/ztest_ft[,"sample1"])+(1/ztest_ft[,"sample2"]))))

### average the z statistic over 15 SNPs for each chromosome seperately
ztest_ft[(which(mbfFT[,1] == "chr1")),9] = rollapply(data=as.numeric(ztest_ft[(which(mbfFT[,1] ==
"chr1")),8]),width=15,FUN=mean,fill=NA)
ztest_ft[(which(mbfFT[,1] == "chr2")),9] = rollapply(data=as.numeric(ztest_ft[(which(mbfFT[,1] ==
"chr2")),8]),width=15,FUN=mean,fill=NA)
ztest_ft[(which(mbfFT[,1] == "chr3")),9] = rollapply(data=as.numeric(ztest_ft[(which(mbfFT[,1] ==
"chr3")),8]),width=15,FUN=mean,fill=NA)
ztest_ft[(which(mbfFT[,1] == "chr4")),9] = rollapply(data=as.numeric(ztest_ft[(which(mbfFT[,1] ==
"chr4")),8]),width=15,FUN=mean,fill=NA)
ztest_ft[(which(mbfFT[,1] == "chr5")),9] = rollapply(data=as.numeric(ztest_ft[(which(mbfFT[,1] ==
"chr5")),8]),width=15,FUN=mean,fill=NA)
ztest_ft[(which(mbfFT[,1] == "chr6")),9] = rollapply(data=as.numeric(ztest_ft[(which(mbfFT[,1] ==
"chr6")),8]),width=15,FUN=mean,fill=NA)
ztest_ft[(which(mbfFT[,1] == "chr7")),9] = rollapply(data=as.numeric(ztest_ft[(which(mbfFT[,1] ==
"chr7")),8]),width=15,FUN=mean,fill=NA)
ztest_ft[(which(mbfFT[,1] == "chr8")),9] = rollapply(data=as.numeric(ztest_ft[(which(mbfFT[,1] ==
"chr8")),8]),width=15,FUN=mean,fill=NA)
ztest_ft[(which(mbfFT[,1] == "chr9")),9] = rollapply(data=as.numeric(ztest_ft[(which(mbfFT[,1] ==
"chr9")),8]),width=15,FUN=mean,fill=NA)
ztest_ft[(which(mbfFT[,1] == "chr10")),9] = rollapply(data=as.numeric(ztest_ft[(which(mbfFT[,1] ==
"chr10")),8]),width=15,FUN=mean,fill=NA)
```

```
### push all statistics to one tail for calculation of p-value
ztest_ft[,10] = -(abs(ztest_ft[,9]))

### using two-tail test calculate p-value based on normal distribution of mean=0 standard deviation=1
ztest_ft[,11] = 2*(pnorm(ztest_ft[,10],mean=0,sd=1))
### take -log of p-value
ztest_ft[,12] = -(log10(ztest_ft[,11]))
### store chromosome number and consecutive positions.
ztest_ft[,13] = as.character(mbfFT[,1])
ztest_ft[,14] = mbfFT[,15]

############################################################
##### getting directionality of significant regions
##### isolate all SNPs with -log10(p-value) greater then threshold
ft_regions = ztest_ft[which(as.numeric(ztest_ft[,12]) >= 3.35),]
peaks = 1

### number each of the regions allowing for 5Mb gap between significant regions
for(z in 2:length(ft_regions[,1])) {

        if((as.numeric(ft_regions[z,"consec"])-as.numeric(ft_regions[(z-1),"consec"])) > (5*10^6)) {
        peaks = c(peaks,(peaks[(z-1)]+1))
        }

        if((as.numeric(ft_regions[z,"consec"])-as.numeric(ft_regions[(z-1),"consec"])) <= (5*10^6)) {
        peaks = c(peaks,peaks[(z-1)])
        }

}

### append peak number to region matrix and subset pertinent information
ft_regions = cbind(ft_regions,peaks)
plotting_ft_regions = ft_regions[,c(13,1,15)]

### create new matrix for storing region boundaries
regionBoundFt = matrix(ncol=6,nrow=max(as.numeric(plotting_ft_regions[,3])))
colnames(regionBoundFt) = c("Peak","Chr","Left","Right","Length","NumGenes")

for(t in 1:max(as.numeric(plotting_ft_regions[,3]))) {
        ### number the region
        regionBoundFt[t,1] = t
        ### call the chromosome
        regionBoundFt[t,2] = plotting_ft_regions[which(plotting_ft_regions[,2] ==
                min(as.numeric(plotting_ft_regions[which(plotting_ft_regions[,3] == t),2]))),1]
        ### give the left and right boundary
        regionBoundFt[t,3] = ztest_ft[(which(ztest_ft[,1] == min(as.numeric(plotting_ft_regions[which(plotting_ft_regions[,3]
                == t),2])) & ztest_ft[,13] == regionBoundFt[t,2]))-7,"Pos"]
        regionBoundFt[t,4] = ztest_ft[(which(ztest_ft[,1] == max(as.numeric(plotting_ft_regions[which(plotting_ft_regions[,3]
                == t),2])) & ztest_ft[,13] == regionBoundFt[t,2]))+7,"Pos"]
}

### calculate the length of the region
regionBoundFt[,5] = as.numeric(regionBoundFt[,4]) - as.numeric(regionBoundFt[,3])

### create a matrix to store the directionality of the region and significant SNP
FTdirection = matrix(ncol = 2, nrow = nrow(regionBoundFt))
colnames(FTdirection) = c("Direction","SigSNP")
```

```
for(i in 1:nrow(regionBoundFt)) {
	### subset the ztest matrix by the left and right boundary and return the frequency difference
	newsub = ztest_ft[which(ztest_ft[,"chr"] == regionBoundFt[i,"Chr"] &
		as.numeric(ztest_ft[,"Pos"]) >= as.numeric(regionBoundFt[i,"Left"]) &
		as.numeric(ztest_ft[,"Pos"]) <= as.numeric(regionBoundFt[i,"Right"])),"B73.Freq.Diff"]
	### subset the ztest matrix by the left and right boundary and return -log10 pvalue
	newsub2 = ztest_ft[which(ztest_ft[,"chr"] == regionBoundFt[i,"Chr"] &
		as.numeric(ztest_ft[,"Pos"]) >= as.numeric(regionBoundFt[i,"Left"]) &
		as.numeric(ztest_ft[,"Pos"]) <= as.numeric(regionBoundFt[i,"Right"])),"-log10(P_value)"]
	### create new value to give the mean direction of a region and name the contributing parent
	direct = mean(as.numeric(as.character(newsub)))
	if (direct < 0) {
		FTdirection[i,1] = "Mo17"
	}
	if (direct > 0) {
		FTdirection[i,1] = "B73"
	}
	### return the SNP with the highest log10(p-value)
	FTdirection[i,2] = as.numeric(as.character(ztest_ft[which(ztest_ft[,"chr"] == regionBoundFt[i,"Chr"] &
	 as.numeric(ztest_ft[,12]) == max(as.numeric(newsub2))),1]))

}

### append the region boundary matrix the the direction matrix
regionBoundFt = cbind(regionBoundFt, FTdirection)
### save the matrix as a new file.
write.csv(regionBoundFt,"regionBoundFt.csv",row.names=F,quotes=F)

##########################################################################################################
########## repeat with plant height
########## annotation is the same as above for flowering time.
##########################################################################################################
## z = (p_best - p_worst) / sqrt(p_hat*(1-p_hat)*((1/n1)+(1/n2)))
## p_hat = (x1 + x2) / (n1 + n2)
### we could use p_hat = 0.5 (expected allele frequency)
  # yielding : sqrt((((0.5)(0.5))/2N1) + (((0.5)(0.5))/2N2)) as the denominator *bernardo equation

colnames(mbfPH)
ztest_ph = matrix(nrow=length(mbfPH[,1]),ncol=14)
colnames(ztest_ph) = c("Pos","B73.Freq.Diff","count1","count2","sample1",
"sample2","p_hat","Z","Avg_Z","testable_z", "P_value", "-log10(P_value)","chr","consec")
ztest_ph[,1] = mbfPH[,2]
ztest_ph[,2] = mbfPH[,13]-mbfPH[,8]
ztest_ph[,3] = mbfPH[,10]
ztest_ph[,4] = mbfPH[,5]
ztest_ph[,5] = (mbfPH[,10]+mbfPH[,11])
ztest_ph[,6] = (mbfPH[,5]+mbfPH[,6])
ztest_ph[,7] = ((ztest_ph[,"count1"]+ztest_ph[,"count2"])/(ztest_ph[,"sample1"]+ztest_ph[,"sample2"]))
ztest_ph[,8] = (ztest_ph[,2])/(sqrt(
	(ztest_ph[,7])*(1-(ztest_ph[,7]))*((1/ztest_ph[,"sample1"])+(1/ztest_ph[,"sample2"]))))

ztest_ph[(which(mbfPH[,1] == "chr1")),9] = rollapply(data=as.numeric(ztest_ph[(which(mbfPH[,1] ==
"chr1")),8]),width=15,FUN=mean,fill=NA)
ztest_ph[(which(mbfPH[,1] == "chr2")),9] = rollapply(data=as.numeric(ztest_ph[(which(mbfPH[,1] ==
"chr2")),8]),width=15,FUN=mean,fill=NA)
ztest_ph[(which(mbfPH[,1] == "chr3")),9] = rollapply(data=as.numeric(ztest_ph[(which(mbfPH[,1] ==
"chr3")),8]),width=15,FUN=mean,fill=NA)
```

```
ztest_ph[(which(mbfPH[,1] == "chr4")),9] = rollapply(data=as.numeric(ztest_ph[(which(mbfPH[,1] ==
"chr4")),8]),width=15,FUN=mean,fill=NA)
ztest_ph[(which(mbfPH[,1] == "chr5")),9] = rollapply(data=as.numeric(ztest_ph[(which(mbfPH[,1] ==
"chr5")),8]),width=15,FUN=mean,fill=NA)
ztest_ph[(which(mbfPH[,1] == "chr6")),9] = rollapply(data=as.numeric(ztest_ph[(which(mbfPH[,1] ==
"chr6")),8]),width=15,FUN=mean,fill=NA)
ztest_ph[(which(mbfPH[,1] == "chr7")),9] = rollapply(data=as.numeric(ztest_ph[(which(mbfPH[,1] ==
"chr7")),8]),width=15,FUN=mean,fill=NA)
ztest_ph[(which(mbfPH[,1] == "chr8")),9] = rollapply(data=as.numeric(ztest_ph[(which(mbfPH[,1] ==
"chr8")),8]),width=15,FUN=mean,fill=NA)
ztest_ph[(which(mbfPH[,1] == "chr9")),9] = rollapply(data=as.numeric(ztest_ph[(which(mbfPH[,1] ==
"chr9")),8]),width=15,FUN=mean,fill=NA)
ztest_ph[(which(mbfPH[,1] == "chr10")),9] = rollapply(data=as.numeric(ztest_ph[(which(mbfPH[,1] ==
"chr10")),8]),width=15,FUN=mean,fill=NA)

ztest_ph[,10] = -(abs(ztest_ph[,9]))
ztest_ph[,11] = 2*(pnorm(ztest_ph[,10],mean=0,sd=1))
ztest_ph[,12] = -(log10(ztest_ph[,11]))
ztest_ph[,13] = as.character(mbfPH[,1])
ztest_ph[,14] = mbfPH[,15]

###########################################################################################################
### find regions
## ph candidate file is already read in

ph_regions = ztest_ph[which(as.numeric(ztest_ph[,12]) >= 6.34),]
peaks = 1

for(z in 2:length(ph_regions[,1])) {

        if((as.numeric(ph_regions[z,"consec"])-as.numeric(ph_regions[(z-1),"consec"])) > (5*10^6)) {
        peaks = c(peaks,(peaks[(z-1)]+1))
        }

        if((as.numeric(ph_regions[z,"consec"])-as.numeric(ph_regions[(z-1),"consec"])) <= (5*10^6)) {
        peaks = c(peaks,peaks[(z-1)])
        }

}

ph_regions = cbind(ph_regions,peaks)
plotting_ph_regions = ph_regions[,c(13,1,15)]

regionBoundPh = matrix(ncol=6,nrow=max(as.numeric(plotting_ph_regions[,3])))
colnames(regionBoundPh) = c("Peak","Chr","Leph","Right","Length","NumGenes")
for(t in 1:max(as.numeric(plotting_ph_regions[,3]))) {
        regionBoundPh[t,1] = t
        regionBoundPh[t,2] = plotting_ph_regions[which(plotting_ph_regions[,2] ==
                min(as.numeric(plotting_ph_regions[which(plotting_ph_regions[,3] == t),2]))),1]
        regionBoundPh[t,3] = ztest_ph[(which(ztest_ph[,1] ==
                 min(as.numeric(plotting_ph_regions[which(plotting_ph_regions[,3] == t),2])) & ztest_ph[,13] ==
                regionBoundPh[t,2]))-7,"Pos"]
        regionBoundPh[t,4] = ztest_ph[(which(ztest_ph[,1] ==
                max(as.numeric(plotting_ph_regions[which(plotting_ph_regions[,3] == t),2])) & ztest_ph[,13] ==
                regionBoundPh[t,2]))+7,"Pos"]
}

regionBoundPh[,5] = as.numeric(regionBoundPh[,4]) - as.numeric(regionBoundPh[,3])
```

```
hib=c()
for(i in 1:nrow(regionBoundPh)) {

        sub = ztest_ph[which(ztest_ph[,13] == regionBoundPh[i,2]),]
        effect = mean(as.numeric(sub[which(as.numeric(sub[,1]) >= as.numeric(regionBoundPh[i,3])
        & as.numeric(sub[,1]) <= as.numeric(regionBoundPh[i,4]))),2]),na.rm=T)
        hib = c(hib,effect)

}

PHdirection = matrix(ncol = 2, nrow = nrow(regionBoundPh))
colnames(PHdirection) = c("Direction","SigSNP")
for(i in 1:nrow(regionBoundPh)) {
        newsub = ztest_ph[which(ztest_ph[,"chr"] == regionBoundPh[i,"Chr"] &
                as.numeric(ztest_ph[,"Pos"]) >= as.numeric(regionBoundPh[i,"Leph"]) &
                as.numeric(ztest_ph[,"Pos"]) <= as.numeric(regionBoundPh[i,"Right"])),"B73.Freq.Diff"]
        newsub2 = ztest_ph[which(ztest_ph[,"chr"] == regionBoundPh[i,"Chr"] &
                as.numeric(ztest_ph[,"Pos"]) >= as.numeric(regionBoundPh[i,"Leph"]) &
                as.numeric(ztest_ph[,"Pos"]) <= as.numeric(regionBoundPh[i,"Right"])),"-log10(P_value)"]
        direct = mean(as.numeric(as.character(newsub)))
        if (direct < 0) {
                PHdirection[i,1] = "Mo17"
        }
        if (direct > 0) {
                PHdirection[i,1] = "B73"
        }
        PHdirection[i,2] = as.numeric(as.character(ztest_ph[which(ztest_ph[,"chr"] == regionBoundPh[i,"Chr"] &
                as.numeric(ztest_ph[,12]) == max(as.numeric(newsub2))),1]))

}

regionBoundPh = cbind(regionBoundPh, PHdirection)
write.csv(regionBoundPh,"regionBoundPh.csv")
```

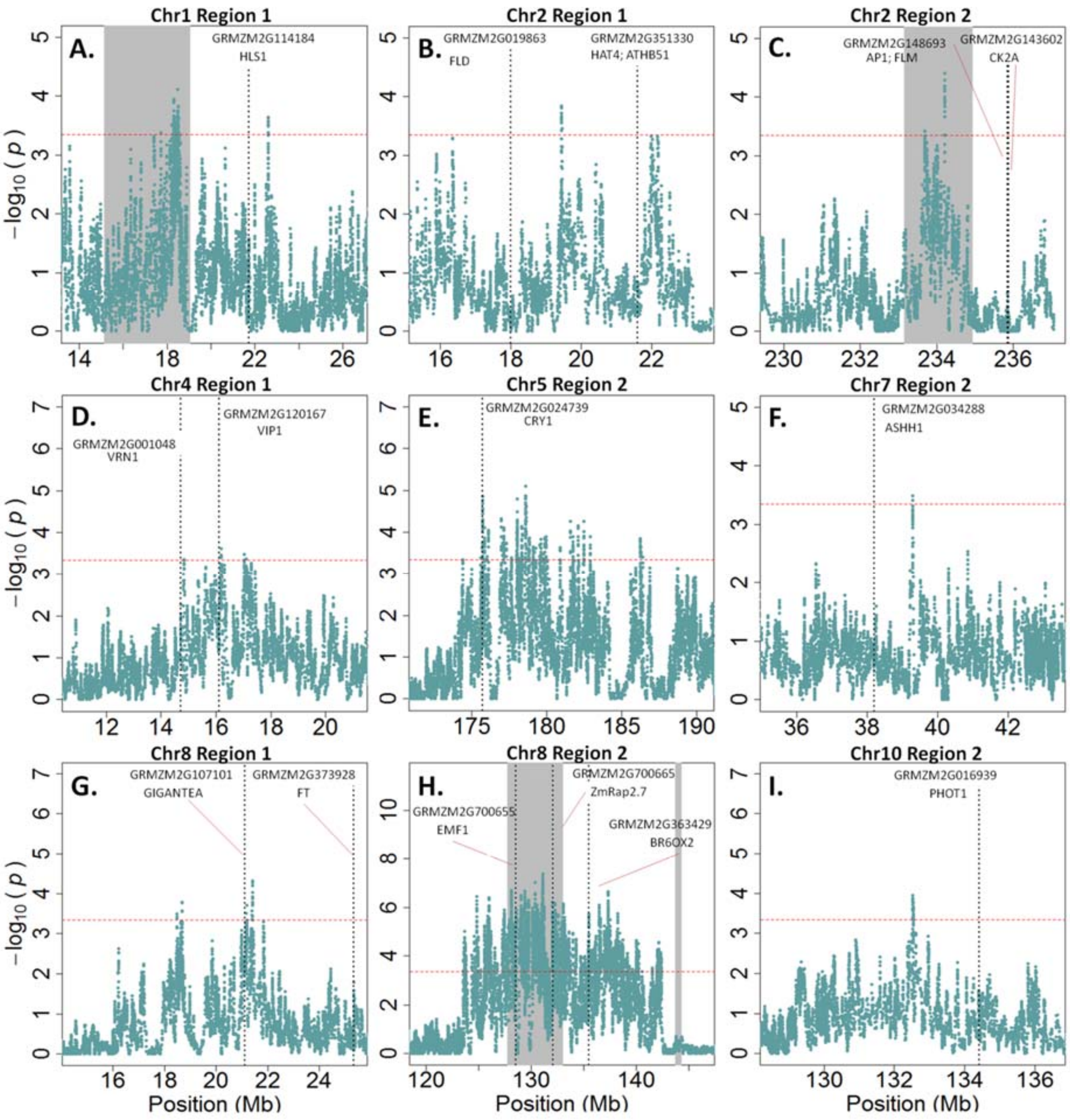

Figure S1 Flowering time candidate gene overlap. Shown are genomic regions identified in the Intermated B73 x Mo17 (IBM) Syn14 and IBM recombinant inbred line populations within 4 Mb of candidate genes for flowering time. Gray shaded regions indicate the 1.5 LOD interval for IBM RIL QTL. The red dotted line indicates a 0.5% empirical outlier threshold for BSA-sequencing.

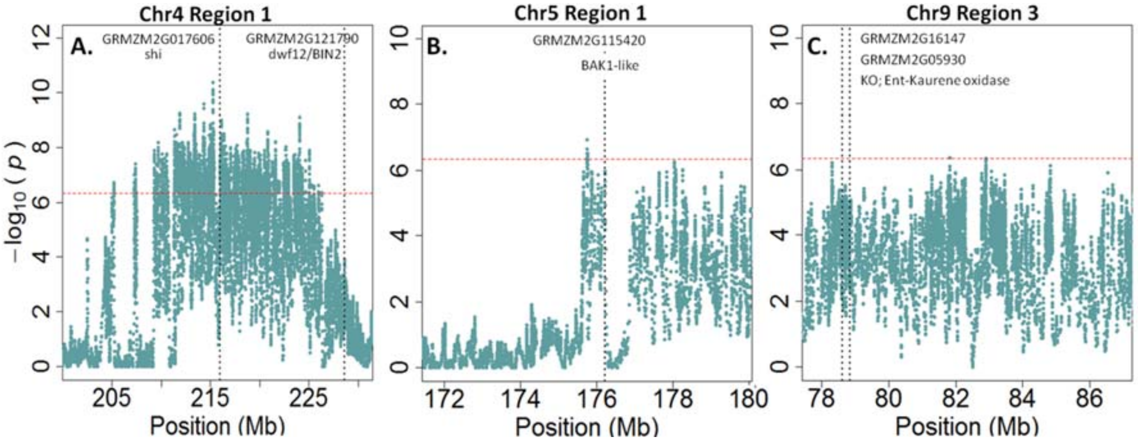

Figure S2 Plant height candidate gene overlap. Shown are genomic regions identified in the Intermated B73 x Mo17 (IBM) Syn14 within 4 Mb of candidate genes for plant height. The red dotted line indicates a 0.5% empirical outlier threshold for BSA-sequencing.
