# Supplementary material for: Shared Genomic Regions Between Derivatives of a Large Segregating Population of Maize Identified Using Bulked Segregant Analysis Sequencing and Traditional Linkage Analysis
Source: G3 (Bethesda). 2015 Jun 1;5(8):1593–602. doi: 10.1534/g3.115.017665 (PMC4528316; doi:10.1534/g3.115.017665)
Supplement: Supporting Information [file supp_g3.115.017665_TableS1.pdf]

Table S1 List of Intermated B73 X Mo17 (IBM) recombinant inbred lines (RILs) and corresponding low density phenotypes. List of IBM RIL genotypes included in the density experiment along with plant height (PH) collected on five individual plants (P1-P5) per plot and flowering time (growing degree days [GDD]) per plot for each RIL when evaluated at a density of 16,500 plants ha^{-1} using two field replications (R1 and R2).

Geno	R1PH1	R1PH2	R1PH3	R1PH4	R1PH5	R1GDD	R2PH1	R2PH2	R2PH3	R2PH4	R2PH5	R2GDD
M0001	150	.	170	180	145	937	.	.	130	135	.	937
M0005	.	180	175	170	180	905	180	185	185	185	185	964
M0007	160	150	165	165	165	825	145	150	150	155	145	825
M0008	.	155	130	155	155	1013	.	160	.	170	150	1040
M0009	170	110	160	165	.	905	190	.	.	.	175	964
M0010	.	.	.	195	180	937	.	185	205	195	190	937
M0012	140	165	145	135	150	876	145	140	.	.	145	825
M0014	.	140	180	150	150	850	.	140	145	150	155	905
M0016	.	75	120	120	120	799	140	140	140	140	140	825
M0017	175	170	185	175	175	876	180	.	170	165	170	825
M0018	.	125	150	130	120	876	125	130	.	115	.	876
M0019	.	160	170	130	165	964	.	165	150	150	.	1013
M0021	160	165	155	165	160	850	155	155	170	.	.	850
M0022	145	.	.	145	165	964	145	145	160	140	.	905
M0023	.	145	140	155	155	905	.	.	125	135	.	850
M0024	200	210	.	200	200	937	195	215	205	200	205	937
M0025	145	140	135	145	145	825	140	155	150	.	.	825
M0026	165	150	170	160	155	905	170	170	165	150	.	905
M0028	150	155	150	155	150	876	160	.	145	.	165	905
M0029	145	140	135	130	145	876	.	160	135	145	165	876
M0030	150	.	170	150	145	1040	.	155	150	160	160	964
M0031	180	170	200	.	160	850	.	.	.	180	190	964
M0032	190	160	165	145	170	937	.	140	185	180	155	989
M0034	130	125	130	145	135	825	125	135	.	125	125	825
M0035	100	130	135	135	.	850	.	150	145	145	130	905
M0036	.	200	180	.	.	937	180	160	180	185	170	937
M0039	180	180	.	.	195	876	195	190	180	.	190	876
M0041	175	195	.	190	.	905	180	180	150	.	150	905
M0042	.	.	155	150	135	964	140	.	150	140	145	876
M0043	120	135	130	.	130	1013	135	.	.	140	150	989
M0045	145	145	160	150	145	825	.	140	145	.	.	876
M0048	.	155	155	170	175	1084	160	.	155	165	.	1121
M0051	.	180	180	175	.	850	190	185	190	170	200	850
M0052	140	140	.	.	135	825	140	150	135	.	135	825
M0054	185	.	190	195	180	825	.	180	.	175	170	850
M0055	.	140	.	.	150	754	130	.	150	145	.	825
M0057	130	.	130	.	155	1121	145	120	120	145	135	1065
M0058	145	155	150	150	150	937	140	140	.	150	150	937
M0059	165	170	170	170	150	876	150	170	155	140	.	876
M0060	150	155	160	150	145	905	140	140	145	120	155	905
M0061	175	165	170	150	155	876	.	175	.	160	155	876
M0063	130	130	135	125	120	876	135	130	140	120	125	905
M0063	.	.	140	125	120	876	130	130	.	.	.	905
M0067	160	165	160	160	180	876	170	170	.	160	160	964
M0068	165	175	.	190	185	905	200	.	165	195	175	905
M0071	145	135	120	.	140	905	135	135	150	140	140	850
M0075	.	145	130	145	150	773	.	155	.	.	135	773
M0075	140	150	150	155	145	799	145	.	135	140	120	754
M0076	165	160	185	165	190	876	190	160	160	.	165	876
M0077	.	170	170	170	155	876	.	.	165	160	160	876
M0079	.	195	175	185	180	964	.	.	185	195	190	1013
M0080	.	165	175	155	160	850	155	160	160	155	175	876
M0081	.	180	180	190	200	964	.	170	175	185	185	964

Table S1 (cont.)

Geno	R1PH1	R1PH2	R1PH3	R1PH4	R1PH5	R1GDD	R2PH1	R2PH2	R2PH3	R2PH4	R2PH5	R2GDD
M0082	165	195	195	170	190	905	190	195	195	.	195	937
M0083	.	.	120	130	140	850	.	.	125	120	130	773
M0085	170	165	165	150	165	876	.	165	160	140	165	905
M0086	.	165	160	140	180	1084	165	160	170	160	160	937
M0087	150	.	.	150	.	825	180	.	165	.	145	773
M0090	140	140	145	155	150	937	150	150	.	.	130	905
M0092	150	135	140	120	140	773	.	135	120	.	125	754
M0097	160	145	155	170	155	799	.	150	150	.	170	825
M0098	.	140	140	140	135	728	150	135	140	130	130	773
M0099	205	.	210	200	215	1065	.	.	170	.	180	1065
M0101	175	170	155	170	135	964	.	150	.	170	140	989
M0105	125	135	130	135	130	905	.	135	130	150	115	905
M0105	.	.	130	135	140	850	.	.	125	.	130	825
M0106	.	190	185	185	.	905	195	200	190	190	190	905
M0109	.	.	.	165	160	876	170	.	170	185	170	937
M0111	160	165	.	165	.	905	.	.	185	180	205	1040
M0114	145	145	135	140	135	825	.	145	140	.	.	825
M0116	.	190	170	205	175	937	170	190	185	.	170	989
M0118	170	170	165	175	160	876	.	165	170	160	165	937
M0119	170	180	175	160	140	876	.	190	190	170	.	876
M0120	155	165	165	150	155	1065	170	85	100	110	120	964
M0121	150	150	160	170	160	937	160	155	175	160	175	989
M0123	125	130	130	135	140	850	120	130	135	140	135	905
M0124	140	135	140	130	200	799	150	150	150	.	.	964
M0125	175	180	155	175	165	905	.	155	.	.	155	937
M0126	185	160	170	160	170	876	175	165	170	145	.	850
M0127	165	170	140	.	150	850	140	.	155	160	160	937
M0128	155	180	180	185	195	964	.	190	170	160	180	937
M0128	175	.	190	160	180	905	190	190	165	175	.	937
M0129	180	135	160	175	145	937	.	.	125	125	.	905
M0131	.	155	125	140	160	850	160	150	.	140	160	850
M0132	140	130	135	135	135	773	130	.	145	140	135	773
M0133	100	130	145	125	120	773	.	155	120	150	130	876
M0134	195	170	.	165	195	964	190	150	.	180	160	964
M0138	145	115	140	145	135	799	165	165	.	150	165	876
M0141	.	190	180	205	.	1084	190	195	205	.	190	1084
M0142	110	115	.	.	.	905	.	.	115	130	145	825
M0143	155	170	.	155	.	937	.	170	155	150	.	964
M0145	145	155	.	.	175	905	.	.	140	160	.	876
M0146	175	155	165	170	160	876	.	165	160	160	.	850
M0147	175	.	160	170	160	850	190	175	170	155	165	876
M0150	150	140	145	140	.	850	.	.	.	135	150	850
M0151	160	160	165	165	.	964	.	160	155	.	.	989
M0153	135	125	125	.	150	825	.	145	155	145	140	850
M0154	.	175	170	170	155	876	180	170	.	175	175	799
M0156	140	145	145	150	140	825	160	150	160	155	150	825
M0159	160	160	.	.	160	1065	.	160	165	155	155	1013
M0160	190	185	.	190	145	1013	190	.	180	190	195	989
M0161	.	215	220	165	215	989	200	.	215	205	205	1065
M0161	215	.	220	210	210	989	210	210	215	185	210	1013
M0162	205	.	200	210	210	989	180	205	200	205	200	1013
M0163	.	145	150	150	150	876	.	145	160	185	150	876
M0164	.	140	150	160	160	1040	165	170	.	150	170	1040
M0165	140	170	160	150	150	825	155	165	140	150	.	825
M0167	150	160	.	.	.	876	170	145	150	140	140	825
M0168	150	140	150	.	.	964	150	150	140	150	155	905

Table S1 (cont.)

Geno	R1PH1	R1PH2	R1PH3	R1PH4	R1PH5	R1GDD	R2PH1	R2PH2	R2PH3	R2PH4	R2PH5	R2GDD
M0169	140	150	160	155	.	825	175	150	145	160	125	825
M0171	155	150	150	170	150	825	160	185	185	160	165	876
M0172	145	160	.	150	.	876	.	.	160	165	150	905
M0174	170	185	190	180	170	905	.	.	165	185	175	937
M0174	.	190	210	200	190	937	170	180	185	180	175	937
M0176	140	140	150	165	150	850	150	140	140	.	155	905
M0177	185	175	180	180	180	964	190	.	.	.	180	964
M0178	.	125	125	150	.	876	.	125	140	125	145	937
M0180	160	.	155	135	125	876	175	170	160	170	165	850
M0181	.	.	170	145	160	964	165	.	.	.	180	937
M0182	190	.	185	165	160	825	190	185	180	.	170	850
M0185	165	175	175	175	170	964	170	155	160	160	185	937
M0186	.	170	165	170	160	850	170	175	150	170	170	876
M0187	135	.	130	130	135	876	140	.	.	135	145	876
M0189	175	180	180	165	190	937	.	180	165	185	175	876
M0190	155	180	165	165	160	876	170	155	160	165	150	825
M0191	150	165	170	165	145	905	.	140	180	140	190	937
M0192	150	.	.	175	160	1040	140	140	.	150	.	937
M0194	150	145	130	140	140	937	130	135	140	155	145	937
M0195	.	165	175	180	160	937	160	.	.	.	175	989
M0196	.	.	.	170	160	773	175	190	180	180	185	825
M0197	130	90	.	95	120	989	125	95	95	125	.	964
M0198	175	185	195	180	165	876	185	.	190	185	220	905
M0198	.	185	185	205	190	876	190	200	180	180	195	905
M0199	.	170	170	155	145	905	150	160	.	.	.	989
M0200	150	145	145	150	160	937	145	160	150	140	.	937
M0202	135	145	160	.	135	799	145	155	150	150	145	799
M0202	155	145	150	160	130	799	.	140	140	.	.	799
M0205	150	140	140	150	140	825	.	140	130	120	.	850
M0206	.	.	125	140	.	876	150	145	140	140	150	773
M0208	210	210	210	210	.	1013	.	200	175	.	195	1013
M0210	160	160	165	160	160	876	170	180	160	160	.	905
M0214	135	130	150	155	145	989	145	140	140	.	150	905
M0215	190	170	175	185	.	964	180	185	.	185	.	1040
M0216	160	175	170	160	160	876	175	.	175	180	180	876
M0218	200	200	190	200	.	937	.	.	165	190	.	1065
M0219	170	155	180	185	.	876	180	165	170	185	170	850
M0222	130	125	135	135	130	825	140	130	140	120	130	850
M0223	160	160	150	150	150	876	135	155	175	.	205	905
M0224	115	145	140	155	135	799	155	135	.	.	150	876
M0228	145	.	140	145	.	1105	.	.	140	145	140	905
M0229	150	145	140	135	140	905	.	150	180	130	155	905
M0230	160	145	155	.	145	876	.	.	.	150	165	905
M0232	220	190	200	185	165	964	.	.	195	155	160	1013
M0233	160	145	165	180	165	876	.	180	175	175	.	905
M0236	.	140	.	.	110	964	145	135	.	150	155	905
M0236	145	150	.	150	140	905	.	160	150	135	.	964
M0237	190	225	200	.	.	964	.	195	205	205	210	989
M0238	170	170	170	175	190	989	.	175	180	.	185	1084
M0240	160	140	160	165	160	799	.	150	190	155	150	825
M0241	180	155	170	160	.	905	180	175	165	150	160	825
M0244	125	120	130	125	140	825	.	145	125	120	120	876
M0246	175	170	185	200	190	850	195	.	170	195	190	989
M0250	.	.	180	205	185	964	185	155	175	200	160	964
M0253	145	.	.	150	165	964	150	140	145	.	145	825
M0255	135	105	140	120	140	825	130	130	140	130	125	850

Table S1 (cont.)

Geno	R1PH1	R1PH2	R1PH3	R1PH4	R1PH5	R1GDD	R2PH1	R2PH2	R2PH3	R2PH4	R2PH5	R2GDD
M0255	.	.	130	120	130	825	.	.	140	125	.	850
M0256	180	185	185	185	180	937	185	.	175	.	190	964
M0258	140	150	130	140	130	876	130	135	140	135	130	850
M0261	190	.	.	180	.	1105	180	175	190	.	190	1013
M0262	150	160	145	155	.	905	155	140	155	.	150	876
M0263	125	145	140	145	145	850	.	140	135	140	150	825
M0264	155	120	110	120	100	905	115	130	120	120	110	937
M0265	140	140	140	140	140	773	135	145	140	.	125	825
M0266	165	180	150	145	140	773	.	140	145	140	150	876
M0267	.	.	.	120	140	937	.	155	.	155	115	937
M0268	145	140	140	165	140	905	.	160	150	140	150	876
M0269	130	135	140	100	130	876	110	120	125	135	140	825
M0270	170	175	175	180	195	876	185	170	175	170	185	876
M0271	160	145	160	170	180	876	150	140	150	.	145	905
M0274	.	.	135	160	160	825	160	155	.	.	150	937
M0275	165	170	160	130	.	964	135	.	.	145	150	964
M0276	140	155	150	145	160	1013	175	150	145	140	140	964
M0277	165	140	135	145	145	825	130	.	135	.	130	773
M0279	170	175	175	170	.	1040	.	170	195	170	165	1084
M0280	.	.	180	160	195	937	.	165	.	160	155	876
M0281	.	.	185	195	200	1121	185	210	190	205	215	1065
M0282	170	160	165	160	170	850	160	160	170	170	160	850
M0283	160	145	180	175	165	825	160	175	165	170	170	825
M0284	170	180	.	170	195	799	120	160	.	175	.	905
M0287	.	195	205	210	210	989	.	.	210	190	.	964
M0288	165	.	.	.	160	850	.	155	.	.	145	773
M0289	.	.	170	170	180	754	.	180	175	195	170	825
M0295	150	160	160	.	.	905	155	160	.	140	145	876
M0296	170	150	175	.	170	1013	160	140	180	150	155	1040
M0297	150	145	.	145	155	876	150	170	135	.	130	876
M0298	135	135	135	160	.	876	140	155	.	.	160	964
M0300	215	195	175	185	195	964	.	190	195	200	.	989
M0301	.	160	160	175	135	876	175	165	150	155	180	876
M0303	.	.	.	145	.	989	.	.	.	.	.	1084
M0303	120	150	120	110	135	1013	115	.	140	105	.	1065
M0304	.	140	155	155	165	964	165	170	140	.	170	964
M0305	140	140	135	120	150	825	150	.	150	.	115	876
M0307	.	.	145	145	145	825	.	135	140	140	110	876
M0308	160	160	190	170	195	989	.	190	180	155	195	989
M0309	160	165	180	155	.	850	165	.	170	160	.	825
M0310	125	150	150	130	150	937	130	155	125	150	140	905
M0311	135	135	135	140	140	799	140	145	120	140	140	773
M0313	.	160	150	150	135	905	.	145	.	145	135	876
M0314	170	150	.	160	175	1013	170	165	165	.	.	1013
M0317	155	160	160	135	155	964	155	160	120	140	160	964
M0318	.	175	160	190	175	850	170	.	165	150	.	876
M0318	185	180	185	195	180	825	160	180	165	160	175	850
M0321	.	.	.	170	160	964	155	.	175	175	175	964
M0322	170	.	180	165	170	964	.	.	200	200	.	1121
M0323	170	.	175	170	165	850	.	170	185	190	160	905
M0325	210	200	175	200	180	850	.	180	210	185	220	773
M0328	215	195	205	220	.	937	210	.	.	220	210	989
M0329	.	.	150	170	150	905	.	.	160	140	155	876
M0331	180	185	.	175	.	850	190	185	185	195	.	905
M0332	155	155	155	145	145	964	.	160	160	.	.	964
M0334	130	140	110	125	120	825	105	110	.	110	120	754

Table S1 (cont.)

Geno	R1PH1	R1PH2	R1PH3	R1PH4	R1PH5	R1GDD	R2PH1	R2PH2	R2PH3	R2PH4	R2PH5	R2GDD
M0335	180	.	.	160	155	1013	.	160	.	135	.	964
M0335	.	.	160	150	180	937	.	.	170	145	145	1065
M0336	.	180	140	150	150	754	160	155	155	170	155	825
M0337	200	205	200	200	200	876	170	210	200	210	185	876
M0338	165	140	.	120	115	850	175	140	185	180	150	825
M0340	.	.	.	.	.	1013	175	195	145	.	165	1013
M0340	.	170	.	.	.	937	.	.	165	170	180	937
M0341	170	190	155	160	160	964	170	120	.	180	.	1040
M0342	190	180	160	155	165	989	200	210	210	180	200	1040
M0342	185	190	190	185	180	964	175	180	.	180	180	937
M0344	185	175	200	175	.	964	190	140	.	145	175	1040
M0346	.	185	185	.	200	1013	180	190	180	190	.	989
M0349	.	130	.	130	.	937	.	.	.	.	.	1121
M0349	.	.	135	120	140	964	140	130	155	160	135	989
M0351	.	.	.	.	125	1084	.	.	.	.	.	825
M0351	.	.	.	90	140	964	.	.	.	.	.	964
M0352	.	.	135	120	130	825	.	130	115	125	130	773
M0353	.	.	190	175	195	1065	185	180	185	180	185	1065
M0356	160	155	165	165	165	876	.	145	160	145	145	876
M0356	160	.	.	.	160	876	155	145	165	155	155	905
M0357	.	145	155	150	150	876	135	155	145	130	135	937
M0358	.	150	155	180	150	876	155	175	170	.	165	876
M0360	.	160	150	155	150	825	155	140	145	140	.	850
M0365	180	175	165	165	170	876	180	180	175	170	155	876
M0368	170	160	155	160	195	825	.	185	170	170	180	989
