# Supplementary material for: Shared Genomic Regions Between Derivatives of a Large Segregating Population of Maize Identified Using Bulked Segregant Analysis Sequencing and Traditional Linkage Analysis
Source: G3 (Bethesda). 2015 Jun 1;5(8):1593–602. doi: 10.1534/g3.115.017665 (PMC4528316; doi:10.1534/g3.115.017665)
Supplement: Supporting Information [file supp_g3.115.017665_TableS2.pdf]

Table S2 List of Intermated B73 X Mo17 (IBM) recombinant inbred lines (RILs) and corresponding high density phenotypes. List of IBM RIL genotypes included in the density experiment along with plant height (PH) collected on five individual plants (P1-P5) per plot and flowering time (growing degree days [GDD]) per plot for each RIL when evaluated at a density of 49,000 plants ha^{-1} density using two field replications (R1 and R2).

Geno	R1PH1	R1PH2	R1PH3	R1PH4	R1PH5	R1GDD	R2PH1	R2PH2	R2PH3	R2PH4	R2PH5	R2GDD
M0001	185	175	185	190	175	1467	140	140	135	150	150	1467
M0005	.	220	220	235	215	1467	220	210	220	215	200	1435
M0007	160	180	180	180	175	1355	.	.	180	155	155	1355
M0008	195	195	200	180	195	1467	180	180	185	180	160	1519
M0009	160	200	220	170	165	1406	180	180	180	185	170	1467
M0010	230	210	200	210	210	1435	215	215	225	220	220	1467
M0012	175	175	180	180	175	1355	165	165	165	155	145	1406
M0014	175	180	180	170	170	1329	155	160	160	165	180	1355
M0016	135	150	160	160	150	1284	.	.	.	.	.	1258
M0017	190	190	190	200	190	1303	185	180	190	200	185	1303
M0018	.	.	.	.	.	.	145	145	165	150	150	1355
M0019	190	200	185	180	170	1435	210	185	190	185	180	1570
M0021	200	190	185	200	170	1284	190	190	190	200	180	1355
M0022	175	190	190	195	175	1467	190	185	175	180	165	1494
M0023	170	160	160	160	170	1406	170	155	155	160	155	1406
M0024	225	230	230	230	230	1435	225	215	230	215	205	1435
M0025	170	170	180	180	175	1284	170	165	155	160	160	1284
M0026	190	195	175	185	175	1406	195	185	180	170	170	1406
M0028	175	180	170	175	180	1380	175	185	190	185	180	1355
M0029	160	165	175	165	175	1406	180	155	160	170	155	1406
M0030	190	190	195	195	200	1380	165	170	185	180	190	1435
M0031	220	220	220	210	220	1329	205	190	200	185	185	1406
M0032	205	205	210	220	220	1406	195	195	205	190	190	1467
M0034	150	165	160	165	170	1303	160	150	170	170	155	1380
M0035	145	140	165	135	145	1406	145	165	145	160	160	1380
M0036	210	195	195	200	200	1406	205	180	195	175	200	1406
M0039	210	215	210	205	215	1380	205	205	205	200	200	1406
M0041	205	215	215	215	205	1406	205	195	210	195	210	1435
M0042	160	160	165	155	160	1355	160	160	165	160	160	1406
M0043	185	180	185	185	185	1355	190	175	170	170	175	1303
M0045	160	175	175	170	185	1355	145	175	180	160	165	1406
M0048	175	155	150	165	175	1519	175	185	185	175	175	1494
M0051	205	210	195	205	220	1355	220	195	205	205	210	1355
M0052	165	170	170	160	180	1303	150	160	165	175	160	1303
M0054	220	225	210	230	220	1303	200	200	210	210	200	1406
M0055	170	160	155	170	155	1284	155	150	155	150	145	1355
M0057	180	135	125	140	125	1543	155	160	195	165	180	1570
M0058	170	165	190	160	170	1435	170	190	195	160	185	1543
M0059	210	200	205	200	210	1406	185	180	195	195	180	1406
M0060	170	170	165	160	165	1355	175	180	175	180	160	1435
M0061	175	165	180	190	165	1303	180	195	185	170	200	1380
M0063	.	155	140	145	150	1329	160	160	150	150	145	1329
M0063	145	145	135	135	135	1329	165	145	150	145	135	1406
M0067	190	190	200	170	170	1406	180	175	180	170	185	1406
M0068	220	230	240	220	260	1406	220	195	220	185	200	1467
M0071	175	170	160	160	170	1329	165	150	175	165	155	1355
M0075	.	150	175	170	160	1258	160	175	170	165	165	1258
M0075	215	175	165	180	155	1284	160	165	155	165	160	1258
M0076	195	195	185	205	205	1406	215	205	215	210	215	1435
M0077	210	210	205	200	210	1467	180	185	190	180	175	1406
M0079	200	200	200	190	185	1467	205	175	185	195	180	1467
M0080	175	180	175	170	180	1355	155	150	160	165	155	1380
M0081	205	195	210	205	205	1406	195	200	190	190	205	1406

Table S2 (cont.)

Geno	R1PH1	R1PH2	R1PH3	R1PH4	R1PH5	R1GDD	R2PH1	R2PH2	R2PH3	R2PH4	R2PH5	R2GDD
M0082	210	240	240	220	220	1435	215	225	220	210	190	1614
M0083	140	145	140	155	155	1258	140	130	145	155	145	1303
M0085	205	190	200	205	185	1380	180	190	200	200	205	1406
M0086	195	190	190	195	195	1406	195	200	190	200	200	1435
M0087	190	170	180	190	180	1258	180	170	170	175	165	1284
M0090	150	175	160	165	175	1467	150	150	160	160	145	1494
M0092	160	150	145	140	160	1258	175	160	140	145	150	1258
M0097	170	175	180	170	175	1355	175	155	160	160	165	1284
M0098	180	180	185	185	180	1284	170	135	145	160	145	1258
M0099	215	235	215	240	220	1519	230	235	230	230	240	1635
M0101	200	185	200	160	180	1329	190	160	165	160	175	1303
M0105	160	165	160	160	155	1329	170	170	165	150	160	1355
M0105	150	150	165	175	165	1355	145	160	160	175	155	1380
M0106	220	220	230	220	230	1380	225	185	195	200	190	1435
M0109	180	165	180	220	195	1467	200	200	200	220	200	1406
M0111	205	210	180	205	195	1406	185	200	200	190	185	1406
M0114	155	160	170	175	165	1303	165	175	170	165	155	1303
M0116	220	210	205	220	225	1406	205	205	200	210	215	1467
M0118	195	185	185	180	185	1406	190	185	180	195	190	1467
M0119	185	170	185	185	180	1380	.	.	.	190	190	1380
M0120	195	180	190	190	170	1494	160	180	170	180	185	1494
M0121	180	175	170	165	175	1406	190	195	190	185	190	1467
M0123	170	175	160	170	165	1329	155	150	155	150	150	1406
M0124	170	175	170	175	170	1355	155	145	160	150	140	1406
M0125	185	190	190	185	190	1303	185	180	190	195	185	1406
M0126	220	210	200	190	205	1406	180	180	195	180	175	1406
M0127	185	160	180	180	170	1355	180	185	170	165	170	1435
M0128	.	190	200	175	180	1467	195	210	195	195	185	1467
M0128	225	200	190	225	195	1467	185	195	200	215	210	1519
M0129	165	165	180	195	185	1467	190	175	215	180	165	1435
M0131	180	175	155	155	175	1284	160	145	160	165	150	1380
M0132	170	160	165	165	165	1284	170	180	170	165	170	1258
M0133	165	165	165	150	160	1303	160	175	180	160	165	1303
M0134	180	190	210	190	195	1435	190	190	175	185	190	1467
M0138	165	155	155	155	180	1284	165	160	160	165	160	1355
M0141	220	205	195	205	220	1519	205	200	215	210	220	1519
M0142	160	160	155	160	170	1435	150	140	150	140	145	1284
M0143	185	170	150	180	185	1467	170	175	180	160	170	1467
M0145	190	180	170	170	205	1329	165	165	175	175	180	1406
M0146	195	190	205	190	195	1355	190	185	170	180	195	1355
M0147	210	210	195	210	210	1380	190	190	190	180	190	1435
M0150	160	160	165	160	160	1355	165	180	175	170	175	1355
M0151	190	160	190	185	180	1435	160	175	175	180	165	1494
M0153	170	175	175	160	170	1380	170	165	155	170	175	1406
M0154	210	200	200	200	200	1284	200	210	195	195	190	1303
M0156	165	170	175	165	170	1303	175	170	170	180	175	1303
M0159	150	195	170	195	170	1595	160	155	185	165	155	1543
M0160	220	215	220	190	205	1467	200	210	210	190	205	1467
M0161	230	225	230	230	225	1494	235	240	235	240	235	1543
M0161	240	215	240	235	235	1519	220	225	220	225	225	1570
M0162	235	210	230	215	235	1467	220	230	215	225	225	1543
M0163	190	190	185	180	200	1406	185	185	190	190	190	1406
M0164	205	190	185	200	185	1519	195	170	205	205	200	1543
M0165	160	170	170	180	155	1303	180	175	175	170	160	1355
M0167	160	165	160	165	165	1303	165	160	160	160	160	1303
M0168	190	190	195	180	175	1435	170	155	175	160	175	1435

Table S2 (cont.)

Geno	R1PH1	R1PH2	R1PH3	R1PH4	R1PH5	R1GDD	R2PH1	R2PH2	R2PH3	R2PH4	R2PH5	R2GDD
M0169	170	180	175	180	175	1303	180	185	190	190	170	1355
M0171	200	200	200	195	195	1380	185	165	180	205	180	1406
M0172	160	185	185	180	185	1355	185	185	185	175	170	1406
M0174	225	220	230	220	220	1406	205	210	215	220	210	1406
M0174	205	210	210	210	210	1467	195	210	210	200	210	1467
M0176	165	185	190	180	190	1355	170	190	180	155	160	1406
M0177	195	190	180	190	195	1406	210	215	205	200	195	1467
M0178	175	180	170	165	170	1329	150	155	145	135	140	1380
M0180	160	180	170	160	165	1355	180	170	170	180	175	1355
M0181	190	200	200	210	195	1406	180	170	185	190	185	1467
M0182	190	200	200	210	180	1329	205	195	210	205	210	1380
M0185	185	190	195	180	180	1435	210	200	210	215	200	1435
M0186	200	210	200	200	205	1355	195	200	170	180	190	1406
M0187	155	160	170	160	170	1355	155	155	140	150	135	1355
M0189	190	190	195	195	195	1406	190	180	185	175	205	1435
M0190	190	.	185	175	190	1355	190	195	190	190	205	1406
M0191	170	175	165	150	160	1355	190	180	180	180	210	1435
M0192	165	150	170	175	165	1467	175	175	180	180	180	1467
M0194	150	150	165	150	155	1355	145	145	145	160	150	1406
M0195	190	200	195	180	200	1467	200	200	200	200	210	1519
M0196	210	210	240	205	205	1284	210	230	210	210	195	1284
M0197	.	.	.	.	.	1467	115	155	145	115	115	1467
M0198	230	215	220	215	215	1355	205	210	210	215	210	1380
M0198	230	230	230	225	235	1406	210	210	210	210	200	1406
M0199	195	195	195	190	195	1467	185	180	195	175	170	1406
M0200	160	185	170	170	185	1435	160	165	185	185	175	1435
M0202	175	175	150	185	180	1258	170	160	165	165	165	1284
M0202	180	190	175	170	175	1355	150	160	155	160	150	1303
M0205	185	180	185	190	170	1329	160	140	140	145	165	1435
M0206	180	175	180	180	180	1258	160	170	165	155	170	1284
M0208	210	205	215	240	210	1543	215	210	240	240	235	1519
M0210	190	195	190	180	200	1355	180	185	190	190	190	1406
M0214	170	185	160	155	160	1467	175	180	180	175	165	1467
M0215	190	195	190	165	190	1467	205	185	155	180	195	1570
M0216	160	185	195	185	175	1406	175	185	180	180	175	1406
M0218	190	190	195	230	225	1467	225	220	215	220	215	1467
M0219	205	160	195	185	180	1380	175	190	200	200	215	1355
M0222	155	145	155	150	150	1355	160	150	150	165	135	1284
M0223	190	185	190	190	200	1329	170	160	165	180	180	1303
M0224	185	180	170	175	170	1303	170	170	145	165	170	1467
M0228	155	160	135	135	145	1467	150	150	140	150	145	1406
M0229	200	190	190	190	190	1380	170	175	190	175	180	1435
M0230	165	180	175	160	155	1355	170	160	160	160	155	1303
M0232	175	175	220	220	165	1494	220	215	215	230	230	1467
M0233	210	180	200	210	195	1355	195	200	200	200	190	1406
M0236	170	180	175	185	185	1380	170	160	160	165	160	1406
M0236	185	175	175	195	190	1406	155	170	160	160	150	1406
M0237	235	225	230	230	215	1467	235	240	240	200	245	1467
M0238	225	225	225	225	230	1467	205	210	210	200	190	1543
M0240	150	150	175	160	170	1284	175	165	160	180	165	1303
M0241	210	215	210	205	210	1406	160	200	200	220	180	1519
M0244	140	130	150	160	145	1355	140	145	155	130	125	1355
M0246	.	220	205	200	210	1435	200	195	190	190	190	1467
M0250	225	210	210	225	220	1435	220	220	220	225	235	1467
M0253	160	170	165	160	145	1303	160	160	175	165	170	1467

Table S2 (cont.)

Geno	R1PH1	R1PH2	R1PH3	R1PH4	R1PH5	R1GDD	R2PH1	R2PH2	R2PH3	R2PH4	R2PH5	R2GDD
M0255	160	160	160	165	150	1303	135	130	110	130	125	1284
M0255	150	130	165	160	155	1355	165	150	145	140	155	1380
M0256	200	205	210	205	210	1406	200	200	210	190	200	1519
M0258	150	155	165	155	150	1355	145	145	145	145	150	1355
M0261	200	200	195	195	195	1519	190	195	190	180	190	1570
M0262	210	200	195	185	190	1355	185	190	170	165	170	1406
M0263	160	180	180	170	165	1355	170	165	170	165	165	1355
M0264	150	155	150	145	150	1380	130	130	140	130	130	1406
M0265	155	135	150	155	170	1303	140	160	155	155	155	1406
M0266	170	175	170	160	165	1355	170	190	175	175	170	1355
M0267	165	160	165	160	170	1329	135	135	150	150	120	1303
M0268	165	165	155	160	170	1406	165	160	150	140	155	1406
M0269	170	175	170	170	160	1329	160	145	155	155	150	1380
M0270	190	190	195	200	195	1355	185	185	200	205	185	1406
M0271	205	205	195	200	195	1355	190	190	195	185	185	1494
M0274	195	195	200	185	185	1329	180	190	180	190	180	1380
M0275	175	180	170	165	155	1406	165	170	170	175	170	1406
M0276	160	160	180	170	170	1380	175	170	175	155	165	1380
M0277	175	180	190	175	180	1303	180	170	175	165	160	1284
M0279	200	215	210	195	195	1543	.	.	.	140	175	1570
M0280	220	200	225	200	190	1303	205	155	175	200	185	1467
M0281	220	220	230	.	230	1635	200	225	190	200	210	1519
M0282	180	195	180	185	190	1355	180	170	175	180	185	1380
M0283	180	185	195	190	200	1284	205	190	190	190	195	1303
M0284	195	215	200	210	200	1303	190	205	210	210	205	1329
M0287	220	225	225	225	215	1494	210	210	225	220	210	1519
M0288	175	175	165	170	170	1303	205	175	170	165	170	1284
M0289	210	135	200	210	210	1303	200	205	185	195	195	1329
M0295	180	185	180	175	190	1380	175	175	180	185	185	1406
M0296	185	180	180	170	160	1467	185	170	160	170	140	1570
M0297	180	195	180	190	175	1355	175	175	165	170	180	1406
M0298	155	160	160	175	145	1303	155	155	145	140	160	1303
M0300	225	210	220	220	200	1467	185	175	200	225	210	1595
M0301	185	175	150	190	140	1303	160	180	160	160	180	1284
M0303	.	170	145	165	145	1519	160	160	160	160	145	1519
M0303	130	160	155	155	130	1543	175	120	145	170	175	1543
M0304	180	190	185	190	200	1329	175	185	190	180	160	1435
M0305	170	165	170	165	175	1355	170	165	170	175	165	1355
M0307	155	170	180	175	170	1303	180	165	175	165	145	1406
M0308	220	220	185	200	200	1467	210	200	190	190	190	1570
M0309	180	190	190	195	190	1303	200	190	185	210	180	1284
M0310	145	145	150	145	125	1406	165	115	125	130	145	1467
M0311	185	160	165	165	160	1284	160	175	180	165	150	1355
M0313	165	165	160	160	170	1303	155	200	175	170	170	1435
M0314	180	185	175	180	185	1435	190	180	190	190	195	1519
M0317	180	205	195	155	185	1355	175	175	195	175	185	1494
M0318	215	215	220	220	220	1284	.	170	180	205	175	1355
M0318	210	205	205	205	220	1303	190	215	210	190	180	1355
M0321	150	180	170	165	170	1435	190	185	190	195	195	1467
M0322	210	220	205	220	210	1406	210	210	220	220	200	1435
M0323	190	195	155	180	180	1355	195	180	200	190	190	1355
M0325	235	230	240	230	220	1329	220	215	215	240	235	1355
M0328	230	230	230	235	235	1435	230	235	230	230	240	1467
M0329	.	185	160	160	170	1355	.	170	165	280	155	1406
M0331	210	230	220	225	190	1406	205	175	205	195	215	1435

Table S2 (cont.)

Geno	R1PH1	R1PH2	R1PH3	R1PH4	R1PH5	R1GDD	R2PH1	R2PH2	R2PH3	R2PH4	R2PH5	R2GDD
M0332	170	170	170	160	165	1329	150	155	180	160	155	1380
M0334	130	135	135	130	140	1258	130	130	135	140	135	1258
M0335	175	180	180	185	175	1329	180	180	160	170	170	1406
M0335	175	190	205	190	180	1406	190	180	200	180	180	1494
M0336	185	185	190	185	180	1284	180	175	195	190	180	1303
M0337	200	210	210	210	210	1355	195	210	215	215	235	1355
M0338	180	180	170	170	190	1258	185	185	195	175	160	1284
M0340	185	185	185	175	150	1406	190	200	195	200	195	1380
M0340	190	190	205	190	185	1406	185	195	190	210	195	1435
M0341	180	190	185	180	195	1406	165	190	190	170	195	1435
M0342	230	230	230	220	215	1467	220	215	225	220	220	1467
M0342	220	230	220	210	210	1467	225	230	220	220	220	1467
M0344	205	220	190	220	220	1467	205	200	190	200	195	1494
M0346	200	200	205	205	205	1467	.	195	210	200	190	1435
M0349	175	170	160	155	160	1435	170	160	170	190	170	1467
M0349	175	180	180	175	190	1467	165	160	175	185	185	1494
M0351	.	160	120	145	170	1303	140	130	135	155	155	1355
M0351	155	170	165	165	175	1355	150	140	160	165	155	1467
M0352	160	155	155	155	150	1258	145	140	145	150	140	1258
M0353	205	210	225	205	200	1494	190	205	195	200	205	1543
M0356	160	170	175	185	170	1355	170	170	180	175	180	1380
M0356	170	180	170	190	180	1406	170	190	165	165	160	1406
M0357	170	170	145	190	170	1355	155	205	170	175	150	1406
M0358	170	165	175	175	200	1406	180	195	180	205	210	1467
M0360	180	200	190	190	190	1303	185	185	190	180	190	1303
M0365	215	230	215	185	210	1380	200	210	210	205	205	1406
M0368	200	195	200	210	210	1355	190	190	190	180	180	1435
