# Supplementary material for: Shared Genomic Regions Between Derivatives of a Large Segregating Population of Maize Identified Using Bulked Segregant Analysis Sequencing and Traditional Linkage Analysis
Source: G3 (Bethesda). 2015 Jun 1;5(8):1593–602. doi: 10.1534/g3.115.017665 (PMC4528316; doi:10.1534/g3.115.017665)
Supplement: Supporting Information [file supp_g3.115.017665_TableS3.pdf]

Table S3 List of Selected Intermated B73 X Mo17 (IBM) Syn14 plants and corresponding phenotypes. List of 92 selected extremes individuals for both plant height (PH) and flowering time (growing degree days [GDD]) from the IBM Syn14 population grown at density of 16,500 plants ha^{-1}.

**Flowering Time**				**Plant Height**			
Early Flowering Pool		Late Flowering Pool		Short Pool		Tall Pool	
Coordinate	*GDD*	*Coordinate*	*GDD*	*Coordinate*	*Plant Height(cm)*	*Coordinate*	*Plant Height(cm)*
DA_6	528	AR_51	1013	AO_47	85	R_90	260
BI_62	587	BJ_41	1013	CY_35	90	E_91	260
BL_62	587	AY _73	1013	AR_77	95	J_63	260
CH_10	587	AC_79	1040	AY_33	95	T_80	260
BI_8	617	AK_72	1040	BR_29	95	CW_68	260
BK_2	617	AM_9	1040	BT_96	105	Q_84	260
CG_20	617	AS_23	1040	U_5	105	CJ_87	260
CJ_64	617	AT_3	1040	CU_11	110	CO_88	260
CS_73	617	BC_43	1040	U_56	110	DI_80	260
DD_47	617	BE_16	1040	DJ_35	120	Q_58	260
DI_13	617	BE_29	1040	AG_65	125	CO_41	260
DL_96	617	C_15	1040	CK_24	125	DH_56	260
AT_20	645	CM_40	1040	I_11	125	M_90	260
BP_18	645	CN_12	1040	Z_39	130	DE_82	260
BU_5	645	CS_8	1040	AZ_46	135	DG_40	260
BV_1	645	CT_18	1040	BA_29	135	AK_70	265
BY_60	645	E_40	1040	BV_29	135	BL_52	265
CD_4	645	G_39	1040	CX_12	135	C_83	265
CI_3	645	J_38	1040	AB_78	140	C_87	265
CW_18	645	J_86	1040	AD_35	140	CA_51	265
CX_51	645	AH_26	1065	AT_33	140	CC_51	265
V_49	645	AJ_58	1065	AT_43	140	CI_19	265
C_13	675	AK_13	1065	AT_44	140	CI_84	265
CW_45	675	CG_16	1065	BE_24	140	CV_94	265
CU_82	675	CM_56	1065	BE_55	140	CX_59	265
CO_16	675	CO_93	1065	BI_31	140	DE_67	265
CP_14	675	CT_86	1065	BJ_78	140	DG_57	265
CO_64	675	DC_63	1065	BK_30	140	DK_56	265
CY_46	675	F_24	1065	BQ_55	140	E_88	265
BA_78	675	J_78	1065	CK_32	140	F_39	265
CF_25	675	O_48	1065	CV_41	140	J_50	265
CT_1	675	B_39	1084	DC_2	140	X_74	265
CQ_57	675	BI_41	1084	DE_49	140	A_64	270
CA_8	675	CZ_76	1084	O_40	140	BN_85	270
DB_93	675	I_25	1084	P_94	140	CB_88	270
CS_31	675	AT_44	1105	U_39	140	CC_67	270
BV_5	675	BM_31	1105	Z_72	140	CU_75	270
BY_21	675	BR_36	1105	CN_16	145	DA_68	270
DH_26	675	BT_39	1105	V_74	145	DC_55	270
CK_20	675	CC_29	1105	Y_1	145	Q_83	270
AD_36	675	DC_38	1105	CC_4	145	X_60	270
BS_10	675	Y_24	1105	DI_81	145	BW_85	275
CO_8	675	BG_38	1121	DF_48	145	Q_34	275
BD_48	675	BH_55	1121	H_18	145	AE_66	280
DJ_94	675	CK_24	1121	G_78	145	AZ_49	280
A_5	675	AF_3	1158	BP_18	145	D_80	280
