# Supplementary material for: Shared Genomic Regions Between Derivatives of a Large Segregating Population of Maize Identified Using Bulked Segregant Analysis Sequencing and Traditional Linkage Analysis
Source: G3 (Bethesda). 2015 Jun 1;5(8):1593–602. doi: 10.1534/g3.115.017665 (PMC4528316; doi:10.1534/g3.115.017665)
Supplement: Supporting Information [file supp_g3.115.017665_FileS1.pdf]

File S1

Supplemental Method: Annotated R code.

Included is an annotated version of R script used for filtering SNPs, calculating Z' statistic, defining significant regions and directionality of significant regions.

```
### change working directory to read in SNP file
setwd("C:/Users/de Leon Lab/Documents")

### read in SNP file
newmbf =
read.table("B73_Mo17_extreme_allele_freq_matrix_3301371_SNPs_bias_correct_100bp_0.25_0.75_control_with_header.txt",
header=T)

### read in maize chromosome lengths file
## this is a file with bp lengths of maize chromosomes and their cumulative length
setwd("C:/Users/de Leon Lab/Documents/Single_Plant_Files/2012/Sequencing_Data/Resequencing Data")
lengths = read.table("maize_length_v2.txt",header=T)

## creating a vector of consecutive lengths
consec_pos = matrix(nrow = length(newmbf[,1]), ncol = 1)

## storage of positions
## all of the positions in matrix will be added to the cumulative length of their corresponding chromosome
consec_pos[(which(newmbf[,1] == "chr1")),] = newmbf[which(newmbf[,1] == "chr1"),2]

### loop through the remaining chromosomes
for(i in 2:10){
	chromo = paste("chr",i,sep="")
	consec_pos[(which(newmbf[,1] == chromo)),] =
	(newmbf[(which(newmbf[,1] == chromo)),2]) + (lengths[(i-1),3])
}

### attach this new vector to the existing matrix
newmbf = cbind(newmbf, consec_pos)

##########################################################################################################
### new analysis
### parse appropriate columns
## the names of each of the columns is below.
##  [1] "chr"                "pos"                "B73_allele"
##  [4] "Mo17_allele"        "control_coverage"   "control_B73_count"
##  [7] "control_Mo17_count" "control_other_count" "control_B73_freq"
## [10] "control_Mo17_freq"  "control_other_freq" "early_coverage"
## [13] "early_B73_count"    "early_Mo17_count"   "early_other_count"
## [16] "early_B73_freq"     "early_Mo17_freq"    "early_other_freq"
## [19] "late_coverage"      "late_B73_count"     "late_Mo17_count"
## [22] "late_other_count"   "late_B73_freq"      "late_Mo17_freq"
## [25] "late_other_freq"    "short_coverage"     "short_B73_count"
## [28] "short_Mo17_count"   "short_other_count"  "short_B73_freq"
## [31] "short_Mo17_freq"    "short_other_freq"   "tall_coverage"
## [34] "tall_B73_count"     "tall_Mo17_count"    "tall_other_count"
## [37] "tall_B73_freq"      "tall_Mo17_freq"     "tall_other_freq"
## [40] "consec_pos"

### create a subsetted matrix for each trait.
mbfFT = newmbf[,c(1:4,13:17,20:24,40)]
```

```
mbfPH = newmbf[,c(1:4,27:31,34:38,40)]

##### filter matrices by allele count, presence of other alleles, unknown chromosome, and presence of NA's
mbfFT = mbfFT[-(which((mbfFT[,5]+mbfFT[,6]) < 20)),]
mbfFT = mbfFT[-(which((mbfFT[,10]+mbfFT[,11]) < 20)),]

### remove any SNPw with > +1SD of mean
mbfFT = mbfFT[-(which((mbfFT[,5]+mbfFT[,6]) > 60.8)),]
mbfFT = mbfFT[-(which((mbfFT[,10]+mbfFT[,11]) > 60.8)),]


if(length(which(mbfFT[,7] > 0))>0) {
		mbfFT = mbfFT[-which(mbfFT[,7] > 0),]
}

if(length(which(mbfFT[,12] > 0)) > 0) {
		mbfFT = mbfFT[-which(mbfFT[,12] > 0),]
}

##### remove monomorphic SNPs
if(length(which(mbfFT[,9] == 1 & mbfFT[,14] ==1)) > 0) {
mbfFT = mbfFT[-(which(mbfFT[,9] == 1 & mbfFT[,14] == 1)),]
}
if(length(which(mbfFT[,8] == 1 & mbfFT[,13] == 1)) > 0) {
mbfFT = mbfFT[-(which(mbfFT[,8] == 1 & mbfFT[,13] == 1)),]
}

mbfFT = mbfFT[-which(mbfFT[,1] == "chrUNKNOWN"),]
mbfFT = mbfFT[-which(is.na(mbfFT[,5])),]
mbfFT = mbfFT[-which(is.na(mbfFT[,10])),]

##########################################################
##### do the same with the plant height matrix

mbfPH = mbfPH[-(which((mbfPH[,5]+mbfPH[,6]) < 20)),]
mbfPH = mbfPH[-(which((mbfPH[,10]+mbfPH[,11]) < 20)),]

if(length(which(mbfPH[,7] > 0))>0) {
		mbfPH = mbfPH[-which(mbfPH[,7] > 0),]
}

if(length(which(mbfPH[,12] > 0)) > 0) {
		mbfPH = mbfPH[-which(mbfPH[,12] > 0),]
}

##### remove monomorphic SNPs

if(length(which(mbfPH[,9] == 1 & mbfPH[,14] == 1)) > 0) {
mbfPH = mbfPH[-(which(mbfPH[,9] == 1 & mbfPH[,14] == 1)),]
}
if(length(which(mbfPH[,8] ==1 & mbfPH[,13] == 1)) > 0) {
mbfPH = mbfPH[-(which(mbfPH[,8] == 1 & mbfPH[,13] == 1)),]
}

mbfPH = mbfPH[-which(mbfPH[,1] == "chrUNKNOWN"),]
mbfPH = mbfPH[-which(is.na(mbfPH[,5])),]
mbfPH = mbfPH[-which(is.na(mbfPH[,10])),]
```

```
### put the matrices in the correct order
mbfFT = mbfFT[order(mbfFT[,"consec_pos"],decreasing=F),]
mbfPH = mbfPH[order(mbfPH[,"consec_pos"],decreasing=F),]

############################################################

### calculate the statistic accross a group of 15 SNPs
library(zoo)

######### the following is the calculation of the z-stat
## z = (p_best - p_worst) / sqrt(p_hat*(1-p_hat)*((1/n1)+(1/n2)))
## p_hat = (x1 + x2) / (n1 + n2)

### create a new matrix for storing the information
ztest_ft = matrix(nrow=length(mbfFT[,1]),ncol=14)

### name the columns of the new matrix
colnames(ztest_ft) = c("Pos","B73.Freq.Diff","count1","count2","sample1",
"sample2","p_hat","Z","Avg_Z","testable_z", "P_value", "-log10(P_value)","chr","consec")

## store SNP positions
ztest_ft[,1] = mbfFT[,2]
## calculate the allele frequency difference
ztest_ft[,2] = mbfFT[,8]-mbfFT[,13]
### store the B73 allele count for each pool
ztest_ft[,3] = mbfFT[,5]
ztest_ft[,4] = mbfFT[,10]
### store total number of reads in each pool
ztest_ft[,5] = (mbfFT[,5]+mbfFT[,6])
ztest_ft[,6] = (mbfFT[,10]+mbfFT[,11])
### calculate p_hat
ztest_ft[,7] = ((ztest_ft[,"count1"]+ztest_ft[,"count2"])/(ztest_ft[,"sample1"]+ztest_ft[,"sample2"]))
### calculate the z statistic
ztest_ft[,8] = (ztest_ft[,2])/(sqrt(
        (ztest_ft[,7])*(1-(ztest_ft[,7]))*((1/ztest_ft[,"sample1"])+(1/ztest_ft[,"sample2"]))))

### average the z statistic over 15 SNPs for each chromosome seperately
ztest_ft[(which(mbfFT[,1] == "chr1")),9] = rollapply(data=as.numeric(ztest_ft[(which(mbfFT[,1] ==
"chr1")),8]),width=15,FUN=mean,fill=NA)
ztest_ft[(which(mbfFT[,1] == "chr2")),9] = rollapply(data=as.numeric(ztest_ft[(which(mbfFT[,1] ==
"chr2")),8]),width=15,FUN=mean,fill=NA)
ztest_ft[(which(mbfFT[,1] == "chr3")),9] = rollapply(data=as.numeric(ztest_ft[(which(mbfFT[,1] ==
"chr3")),8]),width=15,FUN=mean,fill=NA)
ztest_ft[(which(mbfFT[,1] == "chr4")),9] = rollapply(data=as.numeric(ztest_ft[(which(mbfFT[,1] ==
"chr4")),8]),width=15,FUN=mean,fill=NA)
ztest_ft[(which(mbfFT[,1] == "chr5")),9] = rollapply(data=as.numeric(ztest_ft[(which(mbfFT[,1] ==
"chr5")),8]),width=15,FUN=mean,fill=NA)
ztest_ft[(which(mbfFT[,1] == "chr6")),9] = rollapply(data=as.numeric(ztest_ft[(which(mbfFT[,1] ==
"chr6")),8]),width=15,FUN=mean,fill=NA)
ztest_ft[(which(mbfFT[,1] == "chr7")),9] = rollapply(data=as.numeric(ztest_ft[(which(mbfFT[,1] ==
"chr7")),8]),width=15,FUN=mean,fill=NA)
ztest_ft[(which(mbfFT[,1] == "chr8")),9] = rollapply(data=as.numeric(ztest_ft[(which(mbfFT[,1] ==
"chr8")),8]),width=15,FUN=mean,fill=NA)
ztest_ft[(which(mbfFT[,1] == "chr9")),9] = rollapply(data=as.numeric(ztest_ft[(which(mbfFT[,1] ==
"chr9")),8]),width=15,FUN=mean,fill=NA)
ztest_ft[(which(mbfFT[,1] == "chr10")),9] = rollapply(data=as.numeric(ztest_ft[(which(mbfFT[,1] ==
"chr10")),8]),width=15,FUN=mean,fill=NA)
```

```
### push all statistics to one tail for calculation of p-value
ztest_ft[,10] = -(abs(ztest_ft[,9]))

### using two-tail test calculate p-value based on normal distribution of mean=0 standard deviation=1
ztest_ft[,11] = 2*(pnorm(ztest_ft[,10],mean=0,sd=1))
### take -log of p-value
ztest_ft[,12] = -(log10(ztest_ft[,11]))
### store chromosome number and consecutive positions.
ztest_ft[,13] = as.character(mbfFT[,1])
ztest_ft[,14] = mbfFT[,15]

############################################################
##### getting directionality of significant regions
##### isolate all SNPs with -log10(p-value) greater then threshold
ft_regions = ztest_ft[which(as.numeric(ztest_ft[,12]) >= 3.35),]
peaks = 1

### number each of the regions allowing for 5Mb gap between significant regions
for(z in 2:length(ft_regions[,1])) {

        if((as.numeric(ft_regions[z,"consec"])-as.numeric(ft_regions[(z-1),"consec"])) > (5*10^6)) {
        peaks = c(peaks,(peaks[(z-1)]+1))
        }

        if((as.numeric(ft_regions[z,"consec"])-as.numeric(ft_regions[(z-1),"consec"])) <= (5*10^6)) {
        peaks = c(peaks,peaks[(z-1)])
        }

}

### append peak number to region matrix and subset pertinent information
ft_regions = cbind(ft_regions,peaks)
plotting_ft_regions = ft_regions[,c(13,1,15)]

### create new matrix for storing region boundaries
regionBoundFt = matrix(ncol=6,nrow=max(as.numeric(plotting_ft_regions[,3])))
colnames(regionBoundFt) = c("Peak","Chr","Left","Right","Length","NumGenes")

for(t in 1:max(as.numeric(plotting_ft_regions[,3]))) {
        ### number the region
        regionBoundFt[t,1] = t
        ### call the chromosome
        regionBoundFt[t,2] = plotting_ft_regions[which(plotting_ft_regions[,2] ==
                min(as.numeric(plotting_ft_regions[which(plotting_ft_regions[,3] == t),2]))),1]
        ### give the left and right boundary
        regionBoundFt[t,3] = ztest_ft[(which(ztest_ft[,1] == min(as.numeric(plotting_ft_regions[which(plotting_ft_regions[,3]
                == t),2])) & ztest_ft[,13] == regionBoundFt[t,2]))-7,"Pos"]
        regionBoundFt[t,4] = ztest_ft[(which(ztest_ft[,1] == max(as.numeric(plotting_ft_regions[which(plotting_ft_regions[,3]
                == t),2])) & ztest_ft[,13] == regionBoundFt[t,2]))+7,"Pos"]
}

### calculate the length of the region
regionBoundFt[,5] = as.numeric(regionBoundFt[,4]) - as.numeric(regionBoundFt[,3])

### create a matrix to store the directionality of the region and significant SNP
FTdirection = matrix(ncol = 2, nrow = nrow(regionBoundFt))
colnames(FTdirection) = c("Direction","SigSNP")
```

```
for(i in 1:nrow(regionBoundFt)) {
		### subset the ztest matrix by the left and right boundary and return the frequency difference
		newsub = ztest_ft[which(ztest_ft[,"chr"] == regionBoundFt[i,"Chr"] &
			as.numeric(ztest_ft[,"Pos"]) >= as.numeric(regionBoundFt[i,"Left"]) &
			as.numeric(ztest_ft[,"Pos"]) <= as.numeric(regionBoundFt[i,"Right"])),"B73.Freq.Diff"]
		### subset the ztest matrix by the left and right boundary and return -log10 pvalue
		newsub2 = ztest_ft[which(ztest_ft[,"chr"] == regionBoundFt[i,"Chr"] &
			as.numeric(ztest_ft[,"Pos"]) >= as.numeric(regionBoundFt[i,"Left"]) &
			as.numeric(ztest_ft[,"Pos"]) <= as.numeric(regionBoundFt[i,"Right"])),"-log10(P_value)"]
		### create new value to give the mean direction of a region and name the contributing parent
		direct = mean(as.numeric(as.character(newsub)))
		if (direct < 0) {
			FTdirection[i,1] = "Mo17"
		}
		if (direct > 0) {
			FTdirection[i,1] = "B73"
		}
		### return the SNP with the highest log10(p-value)
		FTdirection[i,2] = as.numeric(as.character(ztest_ft[which(ztest_ft[,"chr"] == regionBoundFt[i,"Chr"] &
		 as.numeric(ztest_ft[,12]) == max(as.numeric(newsub2))),1]))

}

### append the region boundary matrix the the direction matrix
regionBoundFt = cbind(regionBoundFt, FTdirection)
### save the matrix as a new file.
write.csv(regionBoundFt,"regionBoundFt.csv",row.names=F,quotes=F)

#########################################################################################################
########## repeat with plant height
########## annotation is the same as above for flowering time.
#########################################################################################################
## z = (p_best - p_worst) / sqrt(p_hat*(1-p_hat)*((1/n1)+(1/n2)))
## p_hat = (x1 + x2) / (n1 + n2)
### we could use p_hat = 0.5 (expected allele frequency)
  # yielding : sqrt((((0.5)(0.5))/2N1) + (((0.5)(0.5))/2N2)) as the denominator *bernardo equation

colnames(mbfPH)
ztest_ph = matrix(nrow=length(mbfPH[,1]),ncol=14)
colnames(ztest_ph) = c("Pos","B73.Freq.Diff","count1","count2","sample1",
"sample2","p_hat","Z","Avg_Z","testable_z", "P_value", "-log10(P_value)","chr","consec")
ztest_ph[,1] = mbfPH[,2]
ztest_ph[,2] = mbfPH[,13]-mbfPH[,8]
ztest_ph[,3] = mbfPH[,10]
ztest_ph[,4] = mbfPH[,5]
ztest_ph[,5] = (mbfPH[,10]+mbfPH[,11])
ztest_ph[,6] = (mbfPH[,5]+mbfPH[,6])
ztest_ph[,7] = ((ztest_ph[,"count1"]+ztest_ph[,"count2"])/(ztest_ph[,"sample1"]+ztest_ph[,"sample2"]))
ztest_ph[,8] = (ztest_ph[,2])/(sqrt(
		(ztest_ph[,7])*(1-(ztest_ph[,7]))*((1/ztest_ph[,"sample1"])+(1/ztest_ph[,"sample2"]))))

ztest_ph[(which(mbfPH[,1] == "chr1")),9] = rollapply(data=as.numeric(ztest_ph[(which(mbfPH[,1] ==
"chr1")),8]),width=15,FUN=mean,fill=NA)
ztest_ph[(which(mbfPH[,1] == "chr2")),9] = rollapply(data=as.numeric(ztest_ph[(which(mbfPH[,1] ==
"chr2")),8]),width=15,FUN=mean,fill=NA)
ztest_ph[(which(mbfPH[,1] == "chr3")),9] = rollapply(data=as.numeric(ztest_ph[(which(mbfPH[,1] ==
"chr3")),8]),width=15,FUN=mean,fill=NA)
```

```
ztest_ph[(which(mbfPH[,1] == "chr4")),9] = rollapply(data=as.numeric(ztest_ph[(which(mbfPH[,1] ==
"chr4")),8]),width=15,FUN=mean,fill=NA)
ztest_ph[(which(mbfPH[,1] == "chr5")),9] = rollapply(data=as.numeric(ztest_ph[(which(mbfPH[,1] ==
"chr5")),8]),width=15,FUN=mean,fill=NA)
ztest_ph[(which(mbfPH[,1] == "chr6")),9] = rollapply(data=as.numeric(ztest_ph[(which(mbfPH[,1] ==
"chr6")),8]),width=15,FUN=mean,fill=NA)
ztest_ph[(which(mbfPH[,1] == "chr7")),9] = rollapply(data=as.numeric(ztest_ph[(which(mbfPH[,1] ==
"chr7")),8]),width=15,FUN=mean,fill=NA)
ztest_ph[(which(mbfPH[,1] == "chr8")),9] = rollapply(data=as.numeric(ztest_ph[(which(mbfPH[,1] ==
"chr8")),8]),width=15,FUN=mean,fill=NA)
ztest_ph[(which(mbfPH[,1] == "chr9")),9] = rollapply(data=as.numeric(ztest_ph[(which(mbfPH[,1] ==
"chr9")),8]),width=15,FUN=mean,fill=NA)
ztest_ph[(which(mbfPH[,1] == "chr10")),9] = rollapply(data=as.numeric(ztest_ph[(which(mbfPH[,1] ==
"chr10")),8]),width=15,FUN=mean,fill=NA)

ztest_ph[,10] = -(abs(ztest_ph[,9]))
ztest_ph[,11] = 2*(pnorm(ztest_ph[,10],mean=0,sd=1))
ztest_ph[,12] = -(log10(ztest_ph[,11]))
ztest_ph[,13] = as.character(mbfPH[,1])
ztest_ph[,14] = mbfPH[,15]

##############################################################################################################
### find regions
## ph candidate file is already read in

ph_regions = ztest_ph[which(as.numeric(ztest_ph[,12]) >= 6.34),]
peaks = 1

for(z in 2:length(ph_regions[,1])) {

		if((as.numeric(ph_regions[z,"consec"])-as.numeric(ph_regions[(z-1),"consec"])) > (5*10^6)) {
		peaks = c(peaks,(peaks[(z-1)]+1))
		}

		if((as.numeric(ph_regions[z,"consec"])-as.numeric(ph_regions[(z-1),"consec"])) <= (5*10^6)) {
		peaks = c(peaks,peaks[(z-1)])
		}

}

ph_regions = cbind(ph_regions,peaks)
plotting_ph_regions = ph_regions[,c(13,1,15)]

regionBoundPh = matrix(ncol=6,nrow=max(as.numeric(plotting_ph_regions[,3])))
colnames(regionBoundPh) = c("Peak","Chr","Leph","Right","Length","NumGenes")
for(t in 1:max(as.numeric(plotting_ph_regions[,3]))) {
		regionBoundPh[t,1] = t
		regionBoundPh[t,2] = plotting_ph_regions[which(plotting_ph_regions[,2] ==
				min(as.numeric(plotting_ph_regions[which(plotting_ph_regions[,3] == t),2]))),1]
		regionBoundPh[t,3] = ztest_ph[(which(ztest_ph[,1] ==
				 min(as.numeric(plotting_ph_regions[which(plotting_ph_regions[,3] == t),2])) & ztest_ph[,13] ==
				regionBoundPh[t,2]))-7,"Pos"]
		regionBoundPh[t,4] = ztest_ph[(which(ztest_ph[,1] ==
				max(as.numeric(plotting_ph_regions[which(plotting_ph_regions[,3] == t),2])) & ztest_ph[,13] ==
				regionBoundPh[t,2]))+7,"Pos"]
}

regionBoundPh[,5] = as.numeric(regionBoundPh[,4]) - as.numeric(regionBoundPh[,3])
```

```
hib=c()
for(i in 1:nrow(regionBoundPh)) {

		sub = ztest_ph[which(ztest_ph[,13] == regionBoundPh[i,2]),]
		effect = mean(as.numeric(sub[which(as.numeric(sub[,1]) >= as.numeric(regionBoundPh[i,3])
		& as.numeric(sub[,1]) <= as.numeric(regionBoundPh[i,4]))),2]),na.rm=T)
		hib = c(hib,effect)

}

PHdirection = matrix(ncol = 2, nrow = nrow(regionBoundPh))
colnames(PHdirection) = c("Direction","SigSNP")
for(i in 1:nrow(regionBoundPh)) {
		newsub = ztest_ph[which(ztest_ph[,"chr"] == regionBoundPh[i,"Chr"] &
				as.numeric(ztest_ph[,"Pos"]) >= as.numeric(regionBoundPh[i,"Leph"]) &
				as.numeric(ztest_ph[,"Pos"]) <= as.numeric(regionBoundPh[i,"Right"])),"B73.Freq.Diff"]
		newsub2 = ztest_ph[which(ztest_ph[,"chr"] == regionBoundPh[i,"Chr"] &
				as.numeric(ztest_ph[,"Pos"]) >= as.numeric(regionBoundPh[i,"Leph"]) &
				as.numeric(ztest_ph[,"Pos"]) <= as.numeric(regionBoundPh[i,"Right"])),"-log10(P_value)"]
		direct = mean(as.numeric(as.character(newsub)))
		if (direct < 0) {
				PHdirection[i,1] = "Mo17"
		}
		if (direct > 0) {
				PHdirection[i,1] = "B73"
		}
		PHdirection[i,2] = as.numeric(as.character(ztest_ph[which(ztest_ph[,"chr"] == regionBoundPh[i,"Chr"] &
				as.numeric(ztest_ph[,12]) == max(as.numeric(newsub2))),1]))

}

regionBoundPh = cbind(regionBoundPh, PHdirection)
write.csv(regionBoundPh,"regionBoundPh.csv")
```
