# Supplementary material for: Shared Genomic Regions Between Derivatives of a Large Segregating Population of Maize Identified Using Bulked Segregant Analysis Sequencing and Traditional Linkage Analysis
Source: G3 (Bethesda). 2015 Jun 1;5(8):1593–602. doi: 10.1534/g3.115.017665 (PMC4528316; doi:10.1534/g3.115.017665)
Supplement: Supporting Information [file supp_g3.115.017665_FigureS2.pdf]

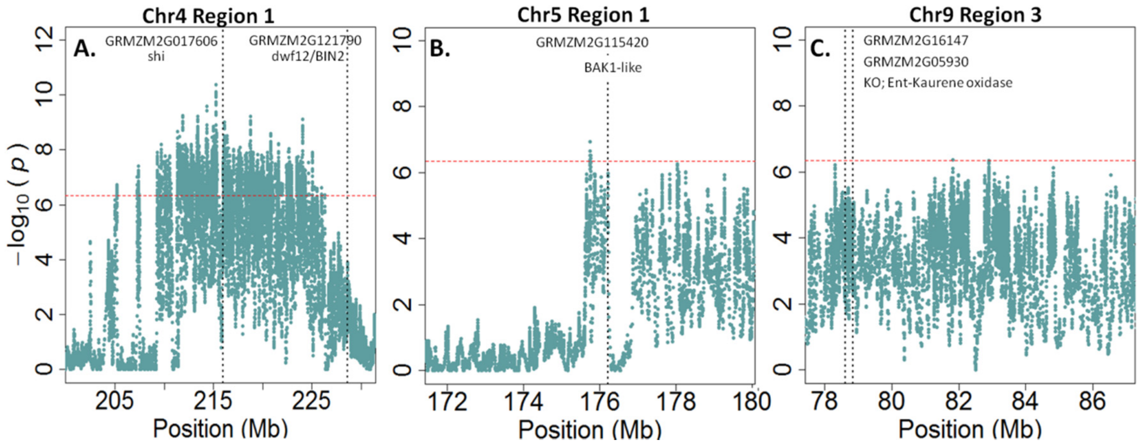

Figure S2 Plant height candidate gene overlap. Shown are genomic regions identified in the Intermated B73 x Mo17 (IBM) Syn14 within 4 Mb of candidate genes for plant height. The red dotted line indicates a 0.5% empirical outlier threshold for BSA-sequencing.
